# Genome-wide association studies of immune, disease and production traits in indigenous chicken ecotypes

**DOI:** 10.1186/s12711-016-0252-7

**Published:** 2016-09-29

**Authors:** Androniki Psifidi, Georgios Banos, Oswald Matika, Takele T. Desta, Judy Bettridge, David A. Hume, Tadelle Dessie, Rob Christley, Paul Wigley, Olivier Hanotte, Pete Kaiser

**Affiliations:** 1 The Roslin Institute and Royal (Dick) School of Veterinary Studies, University of Edinburgh, Easter Bush, Midlothian, EH25 9RG UK; 2 Scotland’s Rural College, Easter Bush, Edinburgh, Midlothian, EH25 9RG UK; 3 School of Life Sciences, University of Nottingham, University Park, Nottingham, NG7 2RD UK; 4 Institute of Infection and Global Health, University of Liverpool, Leahurst Campus, Liverpool, CH64 7TE UK; 5 International Livestock Research Institute, P.O. Box 5689, Addis Ababa, Ethiopia

## Abstract

**Background:**

The majority of chickens in sub-Saharan Africa are indigenous ecotypes, well adapted to the local environment and raised in scavenging production systems. Although they are generally resilient to disease challenge, routine vaccination and biosecurity measures are rarely applied and infectious diseases remain a major cause of mortality and reduced productivity. Management and genetic improvement programmes are hampered by lack of routine data recording. Selective breeding based on genomic technologies may provide the means to enhance sustainability. In this study, we investigated the genetic architecture of antibody response to four major infectious diseases [infectious bursal disease (IBDV), Marek’s disease (MDV), fowl typhoid (SG), fowl cholera (PM)] and resistance to *Eimeria* and cestode parasitism, along with two production traits [body weight and body condition score (BCS)] in two distinct indigenous Ethiopian chicken ecotypes. We conducted variance component analyses, genome-wide association studies, and pathway and selective sweep analyses.

**Results:**

The large majority of birds was found to have antibody titres for all pathogens and were infected with both parasites, suggesting almost universal exposure. We derived significant moderate to high heritabilities for IBDV, MDV and PM antibody titres, cestodes infestation, body weight and BCS. We identified single nucleotide polymorphisms (SNPs) with genome-wide significance for each trait. Based on these associations, we identified for each trait, pathways, networks and functional gene clusters that include plausible candidate genes. Selective sweep analyses revealed a locus on chromosome 18 associated with viral antibody titres and resistance to *Eimeria* parasitism that is within a positive selection signal. We found no significant genetic correlations between production, immune and disease traits, implying that selection for altered antibody response and/or disease resistance will not affect production.

**Conclusions:**

We confirmed the presence of genetic variability and identified SNPs significantly associated with immune, disease and production traits in indigenous village chickens. Results underpin the feasibility of concomitant genetic improvement for enhanced antibody response, resistance to parasitism and productivity within and across indigenous chicken ecotypes.

**Electronic supplementary material:**

The online version of this article (doi:10.1186/s12711-016-0252-7) contains supplementary material, which is available to authorized users.

## Background

Village chickens play an important role in the agriculture of developing countries in sub-Saharan Africa, Asia, Latin America and the Pacific islands (http://www.poultryhub.org/production/backyard-village-poultry/, [1, 2]). The majority (>78 %) of the stock are indigenous ecotypes that are raised in small rural flocks in low input and output (scavenging) management systems [1, 3]. Reports of successful interventions to enhance the productivity of smallholder poultry production in developing countries include control of Newcastle disease in many African countries [4], the poultry distribution scheme in West Bengal, India [5], the use of a hay box brooder in Ethiopia [6], and the Bangladesh BRAC model [7].

Indigenous chickens are well adapted to local production environments. However, due to the relatively low genetic potential and poor levels of management, most birds grow slowly and produce only a few small-sized eggs [1, 8]. Yields from poultry are compromised by extensive losses caused by infectious disease, due to lack of vaccination, biosecurity and other prophylactic measures [1]. The introduction of high-producing exotic birds in Ethiopia has had limited success in rural regions, even when accompanied by farmer training in poultry management, larger flock sizes and increased inputs, likely due to poor adaptation of the introduced breeds to a scavenging production system [9, 10]. Cross-breeding programmes of exotic breeds with indigenous chickens in Ethiopia, Malawi and Kenya have also largely failed due to poor adaptation, uncontrolled mating after the F1 generation and reluctance of local farmers to use exotic birds [1, 11]. Selective breeding and genetic improvement programmes of indigenous ecotypes may provide a sustainable alternative. Since 2006, such programmes have been initiated in Ethiopia, Kenya and Malawi, and have been directed towards a dual-purpose bird with better egg and meat outputs, that is well adapted to village conditions and has the desired morphological traits [9, 11–13]. Current genetic improvement programmes are based on analysis of pedigree and performance records of individual birds. The first results demonstrated the presence of considerable genetic variation in performance traits and strongly suggested that productivity improvement with selective breeding should be possible [9, 11–13]. These selection programmes are yet to consider genetic resistance to major infectious diseases, which could potentially be genetically correlated with production traits [14]. In livestock, including poultry, selection for growth and production traits has been associated with decreased immune function [15, 16]. Furthermore, selection for enhanced disease resistance may reduce growth rate by altering energy partitioning [17]. Conversely, other studies in sheep [18] have shown that more genetically resistant animals may have greater growth; similarly, studies in pigs [19] have shown that animals selected for increased feed efficiency may be less affected by porcine reproductive and respiratory syndrome (PRRS) infection.

Immune response and resistance to infectious diseases are difficult traits to measure and, thereby, improve. Marker-assisted selection or genomic selection might offer effective alternatives to traditional breeding. Nevertheless, genomic technologies for breeding purposes have not yet been applied to indigenous chicken ecotypes in developing countries.

In this study, we investigated the genetic architecture and presence of genetic (co)variability of antibody responses to four major infectious diseases (Marek’s disease, infectious bursal disease, fowl cholera, fowl typhoid), resistance to two parasitic infections (*Eimeria* and cestodes) and two production traits (body weight and body condition score) in two Ethiopian chicken ecotypes (Jarso and Horro), using field-collected data and a high density (580 K) single nucleotide polymorphism (SNP) whole-genome DNA array (Affymetrix^®^ Axiom^®^ HD). We performed variance component analyses, genome-wide association studies (GWAS), and pathway and selective sweep analyses to identify genomic regions controlling disease and production traits, and further investigate the biology of the underlying genetic mechanisms.

## Methods

### Ethical statement

All animal manipulations were conducted in accordance with the revised Animals (Scientific Procedures) Act 1986 with the approval of the University of Liverpool Research Ethics Committee (reference RETH000410).

### Animals

The two indigenous chicken ecotypes used in this study are located in Jarso, in the arid eastern, and Horro, in the sub-humid western part of Ethiopia, two discrete geographical regions about 900 km from each other. The two ecotypes are not phenotypically distinct; similar to other indigenous village chicken populations, there is a wide range of overlapping phenotypic variation within the two populations [20].

### Sampling

Multistage cross-sectional sampling was applied to collect random samples from the two regions. Initially, two market sheds and two villages per market shed were selected within each region. Market sheds are clearly delimited areas comprising villages that rely almost exclusively on a single market for trade. Fifty farms were randomly selected from each village and two chickens over 6 months of age (as estimated by the owner) were randomly selected from each farm. Where possible, one male and one female were chosen, otherwise two females. A total of 760 birds, 376 Jarso and 384 Horro chickens, were sampled in four rounds over 2 years at 6 month intervals, to span the main rainy season [21]. Information on market shed, village, farm, season, sex and age of the birds were recorded [20].

From each bird, freshly-voided faeces were collected to measure *Eimeria* oocysts and cestodes eggs. In addition, a 1.5 mL brachial blood sample was collected into plain tubes flushed with sodium citrate for serological analyses. A drop of blood was also placed on FTA cards for DNA extraction.

### Phenotyping

Individual phenotypes collected for each bird included oocyst counts for coccidiosis (*Eimeria spp*), egg counts for cestodes (any species) and antibody titres for infectious bursal disease (IBDV), Mareks’ disease (MDV), fowl cholera (*Pasteurella multocida*) and fowl typhoid (*Salmonella enterica* serovar Gallinarum). Body weight measured on a sensitive balance and body condition score (BCS) expressed on a 0 to 3 scale [22] were also included in the data.

*Eimeria* oocysts and cestodes eggs in faecal samples were counted with a modified version of the concentration McMaster technique [23]. Due to the small volume of faeces collected from individual birds, we used 1 g of faecal material instead of the recommended 4 g [23].

Antibody titres were based on serological analyses using an in-house enzyme-linked immunosorbent assay (ELISA) for *Salmonella enterica* serovar Gallinarum (SG) and *Pasteurella multocida* (PM), according to the protocol described by Beal et al. [24]. An in-house ELISA based on the protocol of Zelnik et al. [25] was used for MDV. Serological data for IBDV was obtained using a Flockscreen antibody ELISA kit (x-OvO, Dunfermline, UK). All samples were tested in triplicate with a positive and a negative control being added to all plates. In all cases, optical densities (OD) were converted into a ratio to the positive control (s:p ratio) using the following equation [21]:

$${\text{s:p ratio}} = \left( {{\text{mean sample OD}} - {\text{negative control OD}}} \right)/\left( {{\text{positive control OD}} - {\text{negative control OD}}} \right).$$

This data transformation made values comparable between plates by expressing them on a scale where 0 was equal to the negative control and 1 was equal to the positive control. The ELISA plates used for the analyses were also recorded in order to adjust for plate to plate variation in the statistical analyses.

### Genotyping

All birds were genotyped using a 580 K high-density SNP whole-genome DNA array (Affymetrix^®^ Axiom^®^ HD; [26]). This data was subjected to the following quality control thresholds using PLINK v1.07 [27]: minor allele frequency <0.03, call rate <95 %, and Hardy–Weinberg equilibrium (P < 10^−6^). After quality control, 455,463 and 470,486 SNPs were kept for further analyses for Jarso and Horro chicken ecotypes, respectively. Positions of SNPs on the genome were obtained using the Gal-gal4 assembly in Ensemble Genome Browser (www.ensembl.org).

### Genetic parameter estimation

Phenotypes that were significantly skewed (antibody titres of IBDV, MDV, SG and PM, egg counts of *Eimeria* and cestodes, and body weight) were log-transformed in order to normalise their distribution. Genetic parameters were estimated for all traits using a mixed linear univariate model that included the fixed effects of village (1 to 4), market shed (A, B), season [dry (May to July) or rainy (October to November)], sex (male, female), age (6 to 72 months) and ELISA plate (1 to 20, for antibody titres only), and the random genetic effect of the individual bird. Genetic relationships between birds were calculated based on SNP genotypes using the genome-wide efficient mixed model association (GEMMA) algorithm [28] and included in the analyses. Estimates of the obtained variance components were used to estimate the heritability of each trait as the ratio of the additive to the total phenotypic variance. Bivariate analyses were also conducted with the same model to estimate phenotypic and genetic correlations among the studied traits. All above analyses were performed separately for each population (Horro and Jarso) using the ASReml 3.0 software [29].

### Genome-wide association studies

Principal component analysis (PCA) was performed using an identity-by-state matrix based on SNP genotypes to assess genetic differences between the two chicken ecotypes and investigate the presence of population stratification using the GenABEL package of R [30]. The GWAS analyses were performed using the GEMMA algorithm [28], using the same univariate linear mixed model as used for genetic parameter estimation. GWAS analyses were conducted separately for each indigenous population (Jarso and Horro). After Bonferroni correction for multiple testing, significance thresholds were P ≤ 1.1 × 10^−7^ and P ≤ 2.50 × 10^−6^ for genome-wide (P ≤ 0.05) and suggestive (namely one false positive per genome scan) levels, respectively, corresponding to −log_10_(P) of 6.93 and 5.63. In addition, a search for significant SNPs (P ≤ 0.05) after Bonferroni correction at the chromosome-wide level was performed.

Individual significant SNPs in the GWAS were further analysed in a single marker association analysis where the SNP genotype was fitted as a fixed effect into the same univariate mixed model used for genetic parameter estimation. This analysis enabled the estimation of the genetic effect and proportion of variance explained by each SNP as follows: additive effect, a = (AA − BB)/2; dominance effect, d = AB − [(AA + BB)/2]; proportion of phenotypic variance due to SNP = [2pq (a + d(q − p))2]/VP, where, AA, BB and AB represent the predicted trait values for each SNP genotype from the analysis, p and q the SNP allele frequencies and VP the total phenotypic variance of each trait derived without SNP genotypic effects in the model. The extent of linkage disequilibrium (LD) between significant SNPs located on the same chromosome regions was calculated using the r-square statistic of PLINK v1.07 [27].

### SNP and candidate region annotation

All significant SNPs identified in the GWAS were mapped to the reference genome and annotated by using the variant effect predictor (http://www.ensembl.org/Tools/VEP) tool within the Ensembl database and the Gal-gal4 assembly. Moreover, the genes that were located 100 kb upstream and downstream of the significant SNPs were also annotated using the BioMart data mining tool (http://www.ensembl.org/biomart/martview/) within the Ensembl database and the Gal-gal4 assembly. This allowed us to catalogue all the genes that were located in the vicinity of the identified significant SNPs and to create gene lists that contained the genes in the vicinity of all the significant SNPs identified for each disease and production trait. We chose these 200-kb windows based on the average LD calculated previously for the Horro and Jarso chicken populations [31]; in both populations, mild LD (r^2^ ~ 0.2) rarely exceeds 100 kb, while an r^2^ greater than 0.3 did not extend beyond 5 kb.

### Pathway, network and functional enrichment analyses

Identification of potential canonical pathways and networks underlying the candidate genomic regions that were associated with the disease and production traits were performed using the Ingenuity Pathway Analysis (IPA) programme (www.ingenuity.com). IPA constructs multiple possible upstream regulators, pathways and networks that serve as hypotheses for the biological mechanism underlying the phenotypes based on a large-scale causal network derived from the Ingenuity Knowledge Base. Then, IPA infers the most suitable pathways and networks based on their statistical significance, after correcting for a baseline threshold [32]. The IPA score in the constructed networks can be used to rank these networks based on the P-values obtained using Fisher’s exact test [IPA score or P-score = –log10(*P* value)].

The gene lists for each trait were also analysed using the Database for Annotation, Visualization and Integrated Discovery (DAVID; [33]). In order to understand the biological meaning behind these genes, gene ontology (GO) was determined and functional annotation clustering analysis was performed. The *Gallus gallus* background information is available in DAVID and was used for all analyses. The enrichment score (ES) of the DAVID package is a modified Fisher exact P-value calculated by the software, with higher ES reflecting more enriched clusters. An ES greater than 1 means that the functional category is overrepresented.

### Selective sweep analysis

The candidate genomic regions for the disease and the production traits identified in the GWAS analyses were compared with targets of signatures of selection in the same data [31]. For the selective sweep analysis, the SNP data was phased using fastPHASE [34]. Ancestral and derived alleles were assigned using the grey junglefowl (n = 2), green junglefowl (n = 2), Ceylon junglefowl (n = 2), pheasant (n = 3) and Indian peafowl (n = 3) as outgroup populations with allele homozygotes in all outgroup populations defined as the ancestral allele. A signature of selection analysis was performed at the intra-population level with the calculation of integrated haplotype score statistics (iHS) using the R package rehh [34]. This approach identifies SNPs with signals of recent and moderate selection comparing the pattern of linkage disequilibrium at the ancestral and the derived alleles [35]. Moreover, the SweeD software, a likelihood based detection method of selective sweeps based on allele frequencies [36] was used to identify candidate genomic regions that are subjected to strong selection pressure at the population level. Detailed explanation of the methodology that was implemented in this study was previously described in Desta et al. [31].

## Results

### Descriptive statistics

Both the Horro and the Jarso chicken populations demonstrated overwhelming evidence of exposure to all infectious agents studied, which suggests that universal exposure is a reasonable assumption. The mean IBDV antibody titre was higher for Horro birds, whereas MDV, SG and PM antibody titres were similar for birds from the two regions. The higher IBDV antibody titres measured for Horro chickens could be the result of greater exposure to this pathogen in this geographic region and/or stronger host immune response in these chickens. Conversely, the egg counts for *Eimeria* and cestodes parasites were two and four times, respectively, higher for Jarso than for Horro chickens. The higher parasitic load measured for Jarso chickens could be due to higher exposure to the parasites in this geographic region compared to the Horro region and/or to lower host immune response. The average body weight and BCS were marginally higher for Horro chickens. See Additional file 1: Table S1 summarises all measurements for the Horro and Jarso chickens, which demonstrate a clear divergence between the two populations.

### Genetic parameters

We found high heritability estimates (0.75 to 0.79) for IBDV and MDV antibody titres in Horro chickens (Table 1). Moderately high heritability estimates (0.41 to 0.45) were derived for PM antibody titres in Horro and for MDV antibody titres and *Eimeria* and cestodes parasitism in Jarso chickens. The heritability estimates were moderate (0.34 to 0.45) for body weight and BCS in both populations.

**Table 1:** Estimates of variance components and heritabilities for traits studied in Horro and Jarso chickens

	IBDV	MDV	SG	PM	*Eimeria*	Cestodes	BW	BCS
*Horro*								
$\sigma_{\hbox{a}}^{2}$	0.0017	0.18	0.015	0.039	0.007	0.017	0.0027	0.062
$\sigma_{\hbox{p}}^{2}$	0.0021	0.24	0.130	0.094	0.530	0.094	0.0060	0.348
h^2^	0.79	0.75	0.11	0.41	0.01	0.18	0.45	0.18
se	0.15	0.16	0.13	0.16	0.13	0.13	0.15	0.13
*Jarso*								
$\sigma_{\hbox{a}}^{2}$	0.0003	0.0014	0.0096	0.014	0.2559	0.050	0.0025	0.0988
$\sigma_{\hbox{p}}^{2}$	0.0012	0.0030	0.0584	0.086	0.5918	0.14	0.0031	0.2854
h^2^	0.24	0.46	0.17	0.16	0.43	0.35	0.43	0.34
SE	0.12	0.12	0.15	0.14	0.15	0.16	0.12	0.14

*IBDV* log transformed antibody titres measured for infectious bursal disease virus, *MDV* log-transformed antibody titres measured for Mareks’ disease virus, *SG* log-transformed antibody titres measured for *Salmonella enterica* serovan Gallinarum, *PM* log-transformed antibody titres measured for *Pasteurella multocida*; *Eimeria* resistance to *Eimeria* parasitism (log-transformed egg counts/gr of faecal), *Cestodes* resistance to cestodes parasitism (log-transformed egg counts/gr of faecal), *BW* body weight (log-transformed kilograms), *BCS* body condition score (scale 1 to 3); $\upsigma^{2}_{\rm{a}}$ genetic variance, $\upsigma^{2}_{\rm{a}}$ phenotypic variance, *h* ^*2*^ heritability, *SE* standard error

Several significant positive (P < 0.05) phenotypic correlations were detected among the different disease antibody titres and also between *Eimeria* and cestodes parasitism (see Additional file 2: Tables S2 and S3) for details, which implied the presence of infection-interactions. Significant negative phenotypic correlations were estimated between antibody titres and parasite load in the two populations (P < 0.05), e.g., of *Eimeria* with IBDV, SG and PM antibody titres, and between PM and cestodes. These results may suggest immunosuppression in parasitized birds or reciprocal variation in susceptibility.

We found significant positive genetic correlations (P < 0.05) between SG and IBDV antibody titres, between SG and PM antibody titres, and between body weight and BCS in both populations (see Additional file 2: Tables S2 and S3) for details. In addition, a significant positive genetic correlation was estimated between *Eimeria* and cestodes parasitism in Jarso chickens (P < 0.05). There was no significant genetic correlation of the antibody titres to any of the pathogens studied with parasitic diseases, which implies that the negative phenotypic correlations identified above have no genetic basis. Importantly, no significant genetic correlations were found between the disease and production traits.

### Genome-wide association studies

**Fig. 1:**
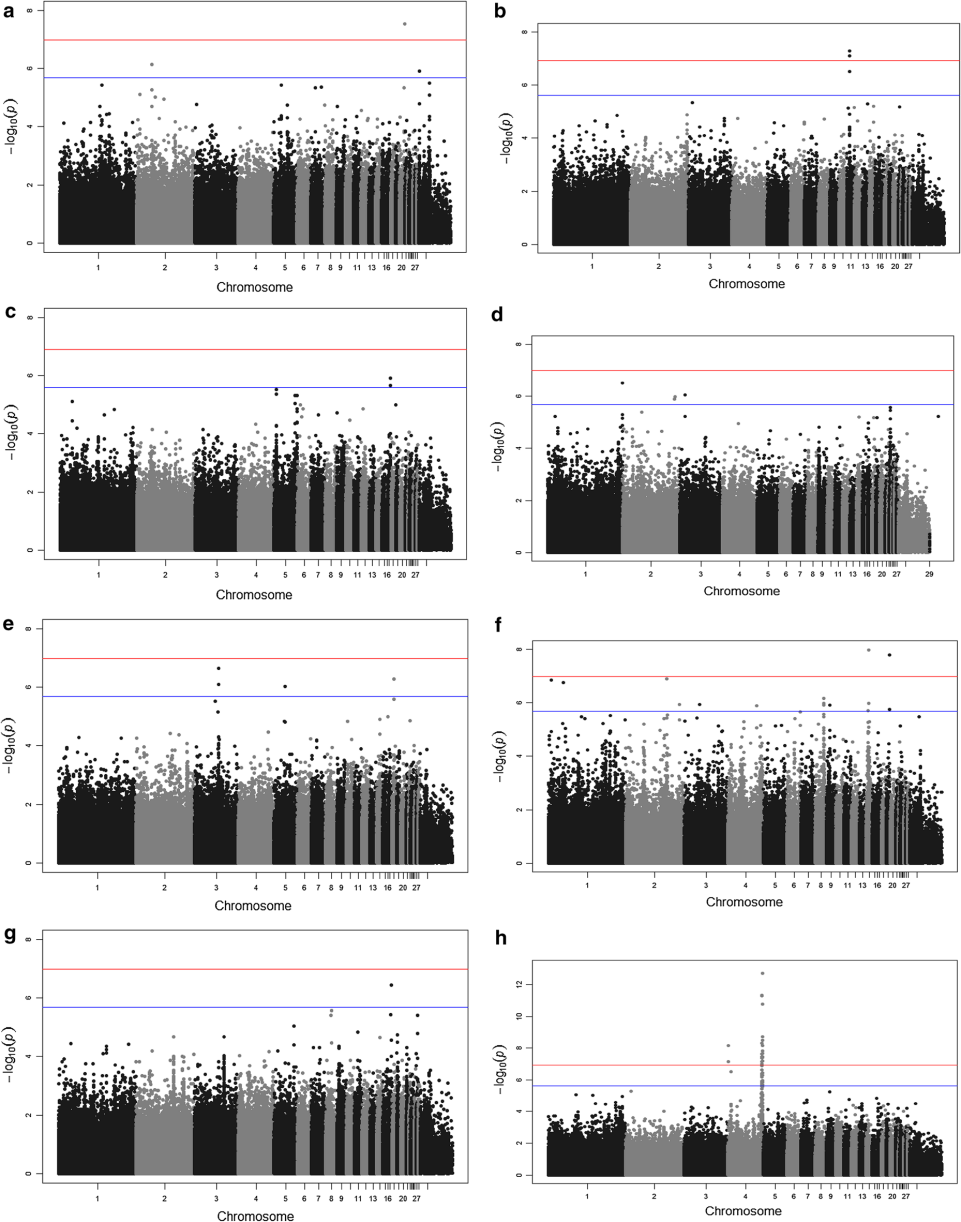
Manhattan plots for the genome-wide association analysis of Jarso chickens. Genomic location (horizontal axis) is plotted against −log_10_(P-value); genome-wide (P < 0.05) and suggestive genome-wide thresholds are shown as *red* and *blue lines*, respectively. Infectious bursal disease virus (IBDV) antibody titre (**a**); Mareks’ disease virus (MDV) antibody titre (**b**); *Salmonella enterica* serovar Galinarum (SG) antibody titre (**c**); *Pasteurella multocida* (PM) antibody titre (**d**); *Eimeria* parasitism resistance (**e**); cestodes parasitism resistance (**f**); body condition score (**g**); body weight (**h**)

**Fig. 2:**
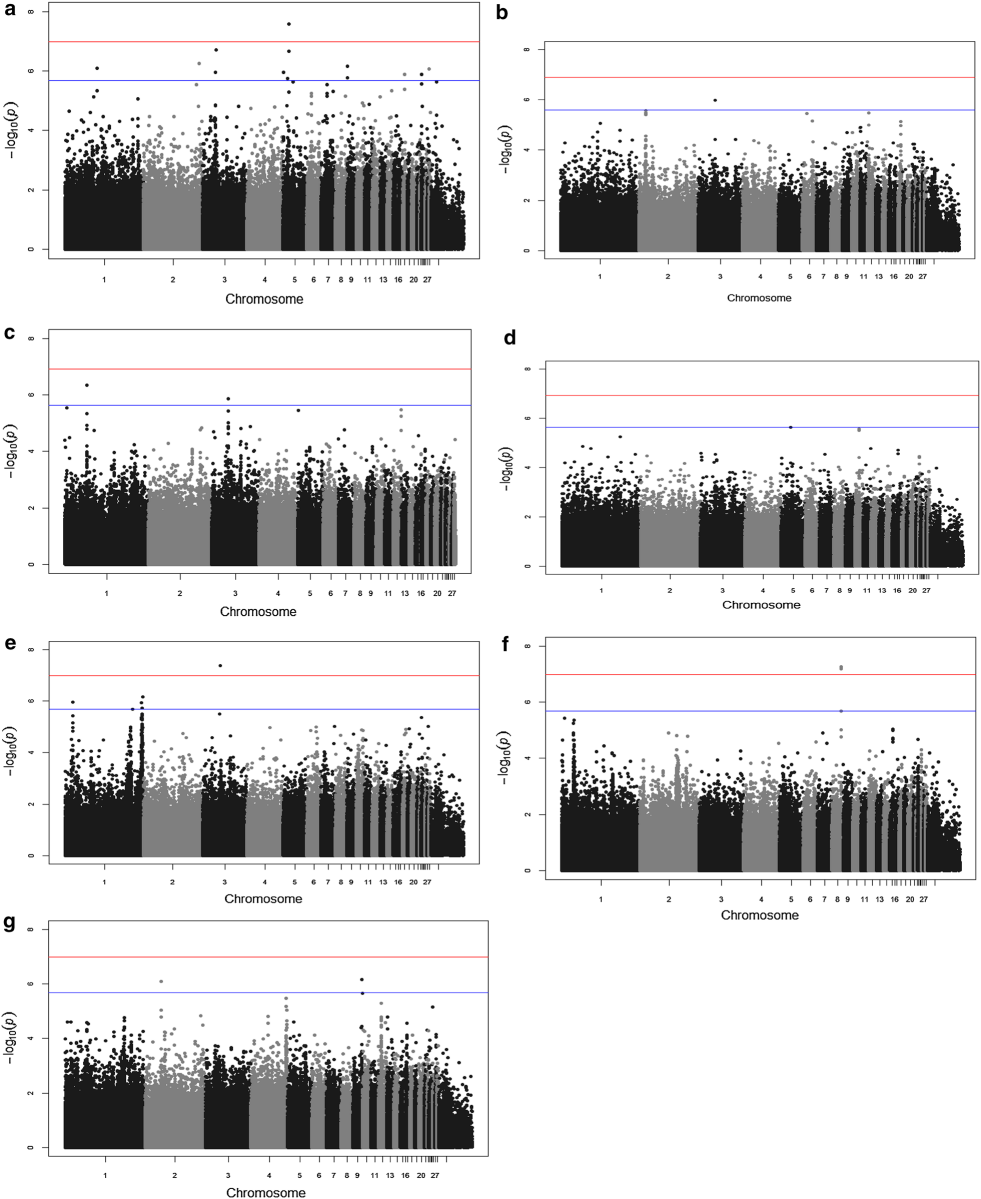
Manhattan plots for the genome-wide association analysis of Horro chickens. Genomic location (horizontal axis) is plotted against −log_10_(P-value); genome-wide (P < 0.05) and suggestive genome-wide thresholds are shown as *red* and *blue lines*, respectively. Infectious bursal disease virus (IBDV) antibody titre (**a**); Mareks’ disease virus (MDV) antibody titre (**b**); *Salmonella enterica* serovar Galinarum (SG) antibody titre (**c**); *Pasteurella multocida* (PM) antibody titre (**d**); cestodes parasitism resistance (**e**); body condition score (**f**); body weight (**g**)

 The PCA analysis identified the two indigenous ecotypes, Jarso and Horro, as genetically distinct groups (see Additional file 3: Figure S1). Therefore, in all subsequent genomic analyses, we treated the two populations separately and found no further evidence of population stratification. In general, GWAS results indicated distinct significant associations for each of the traits in the two ecotypes (see Tables 2, 3; Figs. 1, 2). The Q–Q plots of the GWAS results for Jarso and Horro chickens are in Figures S2 and S3, respectively (see Additional file 4: Figures S2 and S3). We identified genome-wide significant SNPs for antibody titres to IBDV (P-values 2.82 × 10^−8^ to 2.55 × 10^−8^) and cestodes (4.03 × 10^−8^ to 1.80 × 10^−11^) in both ecotypes but on different chromosomes. Moreover, a genome-wide association for MDV antibody titre (P-value of 5.08 × 10^−8^) was also observed but only for Jarso chicken. For the other immune response and disease resistance traits, several SNPs that exceeded the suggestive significance threshold were identified. Regarding production traits, we identified a region on chromosome 4 with 20 SNPs that was associated with body weight at the genome-wide level (6.98 × 10^−8^ to 1.75 × 10^−13^) in the Jarso chickens. The same genomic region was also found to be significant in the Horro chickens, albeit at the chromosome-wide level. Genome-wide significant SNPs were identified on chromosome 8 (6.28 × 10^−8^) for BCS in the Horro chickens, but we did not detect the corresponding genomic region in the Jarso chickens.

**Table 2:** Significant SNPs identified for traits in Jarso chickens

Traits	SNP	Location Chr (bp)	GWAS P-value	Additive effects (P-value)	Dominance effects (P-value)	Phenotypic (%) variance	p	q
IBDV	Affx-51021753* ^,a,b^	*20 (13729523)*	*2.82E−08*	*0.052 (4E−05)*	*−0.032 (0.03)*	*15*	0.04*	*0.96*
	*Affx-50919107* ^a^	*2 (40486311)*	*7.04E−07*	*0.020 (0.04)*	*0.004 (0.15)*	*4*	0.07*	*0.93*
	*Affx-51897552* ^a^	*Z (2805087)*	*1.20E−06*	*−0.005 (0.05)*	*−0.004 (0.28)*	*2*	*0.24*	0.76*
	Affx-51897506	Z (28519476)	3.12*E−*06	*−*0.007 (0.09)	*−*0.000 (0.39)	1	0.18	0.82*
MDV	Affx-50554286* ^,a^	*11 (8097578)*	*5.08E−08*	*0.028 (2E−05)*	*−0.006 (0.31)*	*9*	0.14*	*0.86*
	Affx-50554294* ^,a,b^	*11 (8102461)*	*7.89E−08*	*0.025 (4E−06)*	*−0.005 (0.28)*	*8*	0.26*	*0.74*
	*Affx-50554208* ^a^	*11 (8066972)*	*3.05E−07*	*0.022 (0.01)*	*−0.000 (0.39)*	*5*	0.17*	*0.83*
	Affx-50613892^a,b^	13 (14006092)	5.00*E−*06	0.004 (0.37)	0.032 (0.037)	3	0.09*	0.92
SG	*Affx-50739265* ^a^	*17 (9157484)*	*1.21E−06*	*−0.151 (1E−03)*	*−0.013 (0.38)*	*5*	*0.09*	0.91*
	*Affx-50739250* ^a^	*17 (9151381)*	*2.21E−06*	*−0.134 (3E−03)*	*−0.025 (0.35)*	*4*	*0.10*	0.90*
	Affx-51614950^a^	5 (6690649)	2.99*E−*06	0.078 (1*E−*05)	0.018 (0.31)	4	0.40*	0.60
PM	*Affx-50315740* ^a,b^	*1 (194369733)*	*3.02E−07*	*−0.343 (5E−06)*	*0.219 (0.00)*	*4*	*0.07*	0.93*
	*Affx-51210522* ^a,b^	*3 (13285408)*	*8.75E−07*	*−0.611 (3E−05)*	*0.371 (0.02)*	*9*	*0.03*	0.97*
	*Affx-50856253* ^a,b^	*2 (135976613)*	*1.23E−06*	*−0.323 (1E−05)*	*0.181 (0.03)*	*5*	*0.07*	0.93*
	Affx-51091424^a,b^	23 (3294718)	2.63*E−*06	*−*0.278 (1E*−*08)	0.240 (0.01)	2	0.08	0.92*
	Affx-51891828^a^	Z (22361089)	5.89*E−*06	*−*0.199 (3E*−*03)	0.012 (0.39)	3	0.04	0.96*
*Eimeria*	*Affx-51295947* ^a^	*3 (60276817)*	*2.26E−07*	*0.376 (0.03)*	*0.043 (0.24)*	*3*	*0.07*	0.93*
	*Affx-51295976* ^a^	*3 (60299318)*	*8.09E−07*	*0.342 (0.03)*	*0.032 (0.25)*	*4*	*0.10*	0.90*
	*Affx-50757437* ^a^	*18 (5763355)*	*5.30E−07*	*0.422 (3E−03)*	*0.016 (0.39)*	*7*	*0.14*	0.86*
	*Affx-50757438* ^a^	*18 (5763659)*	*2.61E−06*	*0.426 (4E−04)*	*−0.058 (0.36)*	*7*	*0.12*	0.88*
	*Affx-51550767* ^a^	*5 (28051272)*	*9.20E−07*	*0.654 (9E−05)*	*0.038 (0.19)*	*9*	*0.07*	0.93*
	Affx-50711670^a^	16 (146715)	1.00*E−*05	*−*0.416 (2E*−*03)	0.095 (0.09)	9	0.13*	0.87
Cestodes	Affx-51718143* ^,a,b^	*7 (21664924)*	*1.80E−11*	*0.445 (3E−10)*	*−0.252 (3E−03)*	*10*	*0.04*	0.96*
	Affx-50667122* ^,a,b^	*14 (5803456)*	*1.06E−08*	*0.273 (9E−09)*	*−0.202 (0.03)*	*8*	0.92*	*0.08*
	Affx-50796263* ^,a,b^	*19 (9284997)*	*1.56E−08*	*0.426 (2E−17)*	*−0.411 (2E−05)*	*18*	0.92*	*0.08*
	Affx-50805630* ^,a,b^	*2 (104948558)*	*1.24E−07*	*0.306 (2E−05)*	*−0.151 (0.09)*	*4*	0.96*	*0.04*
	*Affx-50417651* ^a,b^	*1 (7326327)*	*1.39E−07*	*−0.043 (0.02)*	*−0.153 (3E−03)*	*1*	0.90*	*0.10*
	*Affx-50350190* ^a,b^	*1 (37252069)*	*1.71E−07*	*0.445 (3E−10)*	*−0.252 (3E−03)*	*10*	0.96*	*0.04*
	*Affx-51792456* ^a^	*8 (22438049)*	*6.77E−07*	*0.123 (6E−06)*	*−0.051 (0.12)*	*4*	0.76*	*0.24*
	*Affx-51792501* ^a^	*8 (22456586)*	*1.05E−06*	*0.123 (5E−06)*	*−0.052 (0.08)*	*4*	0.77*	*0.23*
	*Affx-51792503* ^a,b^	*8 (22457826)*	*1.21E−06*	*0.114 (5E−07)*	*−0.058 (0.05)*	*4*	0.70*	*0.30*
	*Affx-51875683* ^a,b^	*9 (8596899)*	*1.25E−06*	*0.152 (1E−07)*	*−0.114 (1E−03)*	*6*	0.75*	*0.25*
	*Affx-51474665* ^a^	*4 (72218113)*	*1.32E−06*	*−0.259 (6E−07)*	*NA*	*3*	0.96*	*0.04*
	*Affx-51675956* ^a^	*6 (34224060)*	*2.17E−06*	*0.118 (8E−05)*	*−0.035 (0.26)*	*3*	0.83*	*0.17*
	Affx-51894886^a,b^ Affx-50712683^a^	Z (24758394) 16 (80940)	3.40*E−*06 1.09*E−*04	0.686 (3*E−*15) 0.134 (0.02)	−0.670 (3*E−*11) 0.031 (0.39)	9 5	0.97* 0.87*	0.03 0.13
Bodyweigh	Affx-51502208* ^,a,b^	*4 (87162290)*	*1.75E−13*	*0.051 (4E−13)*	*−0.018 (0.04)*	*25*	0.30*	*0.70*
	Affx-51502179* ^,a^ Affx-51502172* ^,a,b^ Affx-51502191* ^,a,b^ Affx-51502955* ^,a,b^ Affx-51502405* ^,a,b^ Affx-51501548* ^,a,b^ Affx-51501414* ^,a,b^ Affx-51502756* ^,a,b^ Affx-51502383* ^,a,b^ Affx-51501235* ^,a^ Affx-51502311* ^,a^ Affx-51501231* ^,a^ Affx-51502367* ^,a^ Affx-51502246* ^a^ Affx-51500484* ^,a^ Affx-51502298* ^,a^ Affx-51502304* ^,a^ Affx-51501571* ^,a,b^ Affx-51501208* ^,a^	*4 (87149557)* *4 (87146841)* *4 (87154473)* *4 (87546243)* *4 (87266160)* *4 (86818215)* *4 (784066)* *4 (87449896)* *4 (87254896)* *4 (86663849)* *4 (87215318)* *4 (86662441)* *4 (87244290)* *4 (87182268)* *4 (86290949)* *4 (87208267)* *4 (87211838)* *4 (86830246)* *4 (783086)*	*4.56E−12* *5.22E−12* *1.64E−11* *1.88E−09* *3.23E−09* *5.15E−09* *6.88E−09* *7.28E−09* *2.16E−08* *2.32E−08* *3.44E−08* *3.60E−08* *3.86E−08* *4.02E−08* *4.21E−08* *6.33E−08* *6.33E−08* *6.92E−08* *6.98E−08*	*0.057 (5E−10)* *0.051 (4E−13)* *0.060 (6E−10)* *0.033 (0.004)* *0.047 (2E−09)* *0.049(5E−09)* *0.050 (4E−09)* *0.027 (0.002)* *0.046(4E−09)* *0.060 (9E−07)* *0.045 (6E−09)* *0.062 (1E−07)* *0.039 (2E−07)* *0.045 (1E−07)* *0.056 (2E−06)* *0.056 (2E−06)* *0.045 (7E−07)* *0.042 (2E−09)* *0.042 (4E−05)*	*−0.012 (0.19)* *−0.018 (0.04)* *−0.018 (0.09)* *−0.076* *(4E−09)* *−0.018 (0.05)* *−0.026 (0.02)* *−0.027 (0.01)* *−0.059 (3E−08)* *−0.027 (8E−03)* *−0.022 (0.11)* *−0.025 (0.01)* *−0.027 (0.05)* *−0.007 (0.29)* *−0.020 (0.07)* *−0.014 (0.17)* *−0.017 (0.17)* *−0.016 (0.12)* *−0.025 (5E−03)* *−0.008 (0.31)*	*24* *25* *29* *29* *21* *24* *17* *22* *17* *25* *15* *26* *7* *21* *19* *19* *13* *19* *15*	0.22* 0.20* 0.22* 0.13* 0.27* 0.23* 0.24* 0.20* 0.13* 0.26* 0.12* 0.27* 0.13* 0.29* 0.20* 0.13* 0.13* 0.22* 0.30*	*0.78* *0.80* *0.78* *0.87* *0.73* *0.77* *0.76* *0.80* *0.87* *0.74* *0.88* *0.73* *0.87* *0.71* *0.80* *0.87* *0.87* *0.78* *0.70*
BCS	*Affx-50734945* ^a^	*17 (7814709)*	*3.53–07*	*0.290 (6E−05)*	*−0.012 (0.39)*	*7*	*0.86*	0.14*
	Affx-51773927^a^	8 (14332889)	2.73*E−*06	0.195 (6E*−*05)	0.003 (0.39)	6	0.68	0.32*
	Affx-51160997^a,b^	27 (3533019)	2.86*E−*06	0.923 (0.001)	−0.599 (0.03)	9	0.92	0.08*

SNPs with an asterisk * and highlighted in italics are significant at the genome-wide threshold. SNPs in italics are significant at the suggestive genome-wide threshold. SNPs not highlighted are significant at the chromosome-wide threshold

Phenotypic variance:  % proportion of phenotypic variance explained by SNPs; p and q allelic frequencies, with an asterisk * mark the frequencies of the alleles corresponding to high antibody titres for IBDV, MDV, SG, PM, low egg counts for *Eimeria* and cestodes, high body weight and high BCS; *NA* not applicable

*IBDV* infectious bursal disease virus antibody titre, *MDV* Mareks’ disease virus antibody titre, *SG Salmonella enterica* serovan Gallinarum antibody titre, *PM Pasteurella multocida* antibody titre, *Eimeria* resistance to *Eimeria* parasitism, *Cestodes* resistance to cestodes parasitism, *BCS* body condition score

^a^SNPs that had significant additive effects

^b^SNPs that had significant dominance effects

**Table 3:** Significant SNPs identified for traits in Horro chickens

Trait	SNP	Location Chr (bp)	GWAS P-value	Additive effect (P-value)	Dominance effect (P-value)	Phenotypic variance (%)	p	q
IBDV	Affx-5Affx-51526157* ^,a^	*5 (15315358)*	*2.55E−08*	*0.033 (0.05)*	*0.035 (0.09)*	*2*	0.03*	*0.97*
	AfAffx-51242536* ^,a^	*3 (3148207)*	*1.96E−07*	*0.033 (0.01)*	*−0.014 (0.14)*	*10*	0.12*	*0.88*
	*Affx-50862142* ^a,b^	*2 (139341263)*	*5.47E−07*	*0.065 (8E−05)*	*−0.041 (0.02)*	*21*	0.07*	*0.93*
	*Affx-51878048* ^a,b^	*9 (866678)*	*1.68E−06*	*0.0270.04)*	*0.034 (0.05)*	*2*	0.07*	*0.93*
	*Affx-51183095* ^a,b^	*28 (581149)*	*8.47E−07*	*−0.025 (0.04)*	*0.117 (0.01)*	*2*	*0.03*	0.97*
	*Affx-50756295* ^b^	*18 (5404597)*	*1.25E−06*	*−0.003 (0.37)*	*0.032 (0.00)*	*7*	*0.13*	0.87*
	*Affx-51884018* ^a^	*Z (15058127)*	*2.31E−06*	*0.043 (6E−0.4)*	*−0.025 (0.07)*	*12*	0.08*	*0.92*
	Affx-51084536^a,b^	23 *(*1467133)	2.72*E−*06	0.072 *(*0.002)	*−*0.048 *(*0.05)	18	0.04*	0.96
	Affx-50584797^a,b^	12 *(*19824359)	3.88*E−*06	0.025 *(*0.027)	0.000 *(*0.39)	4	0.09*	0.91
MDV	*Affx-51262165* ^a^	*3 (42096244)*	*1.05E−06*	*−0.180 (8E−04)*	*−0.020 (0.37)*	*5*	*0.27*	0.63*
	Affx-50589622^a^	12 *(*3932659)	3.34*E−*06	*−*0.144 *(*5*E−*05)	0.059 *(*0.22)	5	0.35	0.65*
	Affx-50758514^a^	18 *(*6099330)	7.22*E−*06	0.383 *(*5*E−*05)	NA	10	0.10	0.90*
SG	*Affx-50376191* ^b^	*1 (51661206)*	*4.35E−07*	*–0.041 (0.39)*	*0.380 (0.03)*	*2*	*0.05*	0.95*
	*Affx-51254552*	*3 (38112387)*	*1.33E−06*	*−0.032 (0.06)*	*0.025 (0.09)*	*2*	*0.31*	0.69*
	Affx-50583084 Affx-50712674^a^	12 *(*19159478) 16 *(*78709)	3.55*E−*06 1.20*E−*04	0.100 *(*0.08) 0.197 *(*0.02)	*−*0.080 *(*0.14) *−*0.000 *(*0.39)	4 5	0.12* 0.10*	0.88 0.90
PM	*Affx-51540438* ^a^	*5 (22760016)*	*2.31E−06*	*0.081 (9E−04)*	*0.025 (0.29)*	*4*	0.42*	*0.58*
	Affx-50463812^a^	10 *(*10377622)	2.76*E−*06	0.095 (0.01)	0.012 (0.39)	10	0.20*	0.80
	Affx-50463814^a^	10 *(*10378046)	3.19*E−*06	0.08 (0.03)	0.017 (0.38)	9	0.22*	0.78
	Affx-50463818^a^	10 *(*10379061)	3.19*E−*06	0.08 (0.03)	0.017 (0.38)	9	0.22*	0.78
Cestodes	Affx-51266852* ^,a,b^	*3 (44583022)*	*4.03E−08*	*0.289 (7E−06)*	*−0.146 (0.03)*	*11*	*0.15*	0.85*
	*Affx-50313244* ^a,b^	*1 (193416334)*	*6.93E−07*	*0.131 (1E−06)*	*−0.051 (0.15)*	*7*	*0.30*	0.70*
	*Affx-50311899* ^a,b^	*1 (18927296)*	*1.12E−06*	*0.165 (9E−07)*	*−0.096 (0.02)*	*10*	*0.23*	0.77*
	*Affx-51085176* ^a^	*23 (1631142)*	*6.27E−06*	*0.339 (2E−04)*	*−0.180 (0.12)*	*4*	0.07*	*0.93*
Body weight	*Affx-51856375* ^a^	*9 (22119113)*	*6.87E−07*	*−0.044 (0.04)*	*−0.016 (0.32)*	*5*	*0.07*	0.93*
	*Affx-50919051* ^a^	*2 (40447782)*	*7.96E−07*	*−0.041 (1E−06)*	*0.016 (0.11)*	*16*	*0.27*	0.73*
	Affx-51500100^a^	4 *(*86100031)	6.26*E−*06	*−*0.027 *(*1*E−*04)	9*E−*05 *(*0.24)	7	0.62	0.38*
	Affx-50595206^a^	12 *(*6188503)	8.96*E−*06	0.040 *(*0.03)	*−*0.008 *(*0.32)	10	0.19*	0.81
BCS	AfAf fx-51794123* ^,a,^	*8(23157421)*	*6.28E−08*	*−0.224 (2E−07)*	*0.011 (0.39)*	*7*	*0.57*	0.43*
	Affx-51794141* ^,a^	*8 (23164238)*	*6.28E−08*	*−0.224 (2E−07)*	*0.011 (0.39)*	*7*	*0.57*	0.43*
	*Affx-51794116* ^a^	*8 (23154277)*	*2.08E−06*	*−0.193 (2E−05)*	*0.050 (0.24)*	*5*	*0.38*	0.62*
	Affx-50709156^a^	15 (8941550)	8.85*E−*06	0.286 (7*E−*03)	0.057 (0.39)	5	0.13*	0.87

SNPs with an asterisk * and highlighted in italics exceed the genome-wide threshold. SNPs in italics exceed the suggestive genome-wide threshold. SNPs not highlighted exceed the chromosome-wide threshold

Phenotypic variance:  % proportion of phenotypic variance explained by SNPs; p and q allelic frequencies, with an * mark the frequencies of the alleles corresponding to high antibody titres for IBDV, MDV, SG, PM, low egg counts for cestodes, high body weight and high BCS; NA: not applicable

*IBDV* infectious bursal disease virus antibody titre, *MDV* Mareks’ disease virus antibody titre, *SG Salmonella enterica* serovan Gallinarum antibody titre, *PM Pasteurella multocida* antibody titre, *Cestodes* resistance to cestodes parasitism, *BCS* body condition score, *Add* additive effects, *Dom* dominance effects

^a^SNPs that had significant additive effects

^b^SNPs that had significant dominance effects

Several common candidate genomic regions (located within a 0.5-Mb window) were identified within and across the two ecotypes (Tables 2, 3). The common regions were associated with: (1) antibody titres to IBDV and MDV in Horro and *Eimeria* infection in Jarso chickens on chromosome 18 (5.5–6 Mb); (2) antibody titres to PM in Jarso and cestodes infection in Horro chickens on chromosome 1 (193.5–194 Mb); (3) antibody titres to SG in Horro and *Eimeria* and cestodes infections in Jarso chickens on chromosome 16 within the major histocompatibility complex (*MHC*) region; (4) antibody titre to IBDV and cestodes infection in Horro chickens on chromosome 23 (1.4–1.7 Mb); (5) antibody titres to IBDV and SG in Horro chickens on chromosome 12 (12.5 Mb); (6) body weight on chromosome 4 (86.5 Mb) in both chicken ecotypes.

The effects of the significant SNPs identified by the GWAS analyses were mostly additive, explaining 2 to 21 and 1 to 18 % of the phenotypic variance of the disease traits in Horro and Jarso chickens, respectively, and 5 to 15 and 6 to 29 % of the phenotypic variance of the production traits, in Horro and Jarso chickens, respectively (Tables 2, 3). Significant SNPs that were located in the same genomic region were in moderate to high (0.2 to 1) LD with each other (see Additional file 5: Tables S4 and S5) for Jarso and Horro chickens, respectively.

### Annotation of SNPs and candidate regions

The location and annotation of all significant SNPs identified by the GWAS analyses are in see Additional file 5: Tables S4 and S5 for Jarso and Horro chickens, respectively. Most significant SNPs were located in intergenic or intronic regions. However, three of the SNPs that were identified in the Horro chicken data were localized in exonic regions and corresponded to missense deleterious variants. Specifically, Affx-51084536 (associated with IBDV antibody titre) corresponds to a missense variant within the *XK*-*related protein 8* (*XKR8*) gene; Affx-50376191 (associated with SG antibody titre) corresponds to a missense variant within the *myosin*-*9* gene (*MYH9*) gene; and Affx-51266852 (associated with cestodes parasitism) corresponds to a missense variant within the *mitogen*-*activated protein 3 kinase 4* (*MAP3K4*) gene.

Candidate regions were defined as the genomic intervals 100 kb upstream and downstream of the significant SNPs identified by the GWAS and annotated genes within those regions were identified. The lists of candidate genes for all traits are in see Additional file 6: Tables S6 and S7 for Jarso and Horro chickens, respectively. Candidate gene lists contained a few (in total 5 to 40) genes for most traits, with the exception of cestodes resistance in Jarso chickens, which included 80 genes.

### Pathway, network and functional enrichment analyses

Analyses of the candidate genes identified significant pathway enrichment (P < 0.005) (see Additional file 7: Figures S4 and S5). For IBDV, SG and PM antibody titre and for *Eimeria* and cestodes counts enriched pathways were related to innate and adaptive immune responses; antigen presentation, B-cell activating factor, glucocorticoid receptor, complement and primary immunodeficiency signalling were the most common pathways for both ecotypes. For MDV antibody titre, enriched pathways were mostly related with cell cycle regulation but differed for each ecotype. For body weight, enriched pathways were related to pentose phosphate for both ecotypes, which is directly connected with gluconeogenesis and is the major source of NADPH, which is required for anabolic processes [37]. Finally, for BCS, enriched pathways related to inositol and myo-inositol phosphate were identified for the Jarso ecotype.

Several relevant networks were reconstructed from the molecular interactions of the genes that are located in the candidate regions for the immune and disease resistance traits, but most had a moderate (below 30) IPA score (see Additional file 8: Figures S6 and S7). The most informative networks were: (1) the network for cestodes resistance, which included interacting molecules clustered around the *nuclear factor kB* (*NFkB*) gene complex (IPA score = 58); (2) the network for antibody titre to IBDV, with the *v*-*myc avian myelocytomatosis viral oncogene homolog* (*MYC*) gene at the centre (IPA score = 33); and (3) the network for antibody titre to SG, with the interacting molecules clustered around the *NFkB* complex and some classical *map kinases* (*ERK1/2*) (IPA score = 37).

Functional annotation clustering analysis revealed the presence of enriched gene clusters related to antigen processing and presentation and immune response (*Eimeria* and cestodes), regulation of apoptosis (PM), electron transport and oxidation reduction (SG), and regulation of transcription (IBDV) and proteolysis (cestodes) (see Additional file 9: Table S8). Subsequently, putative candidate genes for each trait were selected for the two ecotypes based on their biological function and involvement in pathways, networks and enriched gene clusters of interest. Selected candidate genes as well as the biological processes and the molecular functions involved are presented in Additional file 10: Tables S9 and S10. In general, for antibody responses and resistance to parasitism, most putative candidate genes were immune genes related to innate and adaptive immune response, inflammatory response, metal-ion binding, antigen processing and presentation, interferon secretion, and apoptosis. For the production traits, putative candidate genes were mainly linked with enzymes that are involved in metabolic processes.

### Analysis of selective sweeps

The candidate region on chromosome 18 (5.5–6 Mb) that was associated with antibody titres to IBDV and MDV infections in Horro chickens, and with resistance to *Eimeria* parasitism in Jarso chickens, was within a region that had been previously identified as the site of a selective sweep [31].

## Discussion

Our study set out to investigate the genetic basis of parasitic disease resistance, immune responses to viral and bacterial infections, and production traits of economic importance to indigenous village chickens. Using two indigenous Ethiopian ecotypes, we detected substantial heritable genetic variation and identified genomic regions that affected all studied traits. Putative candidate genes, canonical pathways and networks involved in the underlying molecular mechanisms of antibody response, parasitic disease resistance and production were also identified. Our results should be interpreted in the context of the limitations and advantages of field studies [38, 39]. Compared to controlled challenge experiments, unknown and uncontrolled exposure to infections, may reduce the power of a field study but does not constitute a fatal flaw in demonstrating host genetic differences in resistance [38]. In addition, the natural mixed infections that characterise field studies offer a more realistic picture of the genetic variation and yield results that are more relevant to practical genetic improvement programmes. In our study, we observed a universal exposure to pathogens in all birds during two consecutive years of sampling. A seasonal effect on antibody titres was tested and was not significant. Moreover, no disease outbreaks were recorded during the 2 years of study; therefore, what we report here is the antibody titres to background natural infections in two populations that were raised under local prevailing conditions.

### *Eimeria* and cestodes resistance

Genetic resistance to *Eimeria* has previously been studied in chickens but susceptibility to cestodes parasitism has not. The two genomic regions identified on chromosomes 16 and 18 are located very close to previously reported regions for *Eimeria tenella* resistance in an F2 chicken cross generated between an *Eimeria* resistant indigenous Egyptian line (Fayoumi) and a susceptible Leghorn line [40]. Chromosome 16 in chickens harbours the *MHC*, which encompasses the region that allows T-cells to recognise foreign antigens. The *MHC* region has been the focus of considerable research because of the strong associations between specific *MHC* haplotypes and infectious diseases [41]. Thus, involvement of *MHC* in *Eimeria* and cestodes resistance is not unexpected. The strong positive genetic correlation between *Eimeria* and cestodes resistance suggests the existence of common underlying genetic mechanisms, one of which may be within the *MHC*. Moreover, two of the candidate genes that we identified for cestode resistance, namely the *mitogen*-*activated protein 3 kinase 4* (*MAP3K4*) and *tumour necrosis factor alpha*-*induced protein 1* (*TNFAIP1*) genes, encode proteins that are associated with host immune response to malaria, a disease caused by a parasite that belongs to the same phylum as *Eimeria* [42–44].

### Antibody responses

Our results support previous findings that showed that antibody response is a heritable complex trait. Estimates of heritability for these traits differed between the two populations and ranged from low to high (0.11 to 0.79) with a particularly high heritability for IBDV and MDV for the Horro ecotype. Genome-wide studies of IBDV, MDV, SG and PM antibody titres are very scarce in the literature, especially for indigenous chicken populations. The involvement of specific *MHC* genes in SG and IBDV antibody titres was previously studied using crosses of Fayoumi (indigenous breed) with Leghorn [45] and exotic broilers [46], respectively. In our study, we confirmed the involvement of the *MHC* in SG. Involvement of the *MHC* in the control of antibody response to the other pathogens studied was also anticipated; however LD in this chromosomal region was very low, which suggests a high frequency of recombination and, thus making the detection of associations difficult. Moreover, only a few informative SNPs within the *MHC* region are included in the DNA arrays, which may explain why the role and contribution of this region may be largely undetected.

Some of the significant SNPs identified for antibody titres in our study were located on the sex chromosome Z, which supports previous observations of sex-related differences in immune response and survival rates in chickens [47]. The strong positive genetic correlations that we found among antibody titres to different infectious diseases imply that common underlying genomic regions may exist; thus a breeding programme that would aim at enhancing overall immunocompetence of the birds could be considered. One can reasonably assume that the same programme would also produce birds that respond more efficiently to vaccines. Prior studies in chickens and other species have shown that circulating antibodies may play a role in successful host response to infections. In pigs, Serao et al. [48] found that antibody titres that were measured on sows 46 days after a natural outbreak of PRRS had strong favourable genetic correlations with most liveability traits of litters born during a PRRS outbreak that ranged from −0.72 (number of born mummified) to 0.73 (number of born alive). Increased resistance to *Pasteurella* infections was shown to elicit greater levels of circulating antibodies to important surface antigens in mice, rabbit, cattle and chickens [49–55]. Similarly, chickens selected for high antibody titres to *Escherichia coli*, were also found to have high IBDV antibody titres which conferred increased resistance to IBDV infection [56]. In other diseases, such as *Salmonella*, antibody responses have been shown to play a role in disease resistance [57, 58]. More specifically, in a susceptible mice line after challenge with *Salmonella*, the produced antibodies were of the IgM isotype and remained polyreactive. In contrast, in a line of resistant mice, a specific IgG antibody response that recognised specific bacterial components followed an initial increase of polyreactive IgM antibodies. These results suggest that the synthesis of antibodies directed against *Salmonella* antigens in lines with different susceptibilities may follow distinct pathways [57]. In addition, co-localisation of quantitative trait loci (QTL) for *Salmonella* resistance and *Salmonella* antibody titres have been reported in mice [59] and chickens [60, 61], suggesting that the same QTL modulate both infection resistance and antibody production. Interestingly, the mice lines selected for high antibodies to *Salmonella* antigens showed lower resistance to the disease [59]. On the other hand, *Salmonella* antibody titres have been demonstrated to have a genetic component in chickens, with greater antibody responses being associated with lesser *Salmonella* colonisation, suggesting that enhancement of innate antibody response levels is important [58]. Furthermore, inbred chicken lines with different *MHC* haplotypes and different susceptibilities to IBDV and MDV have been shown to have different antibody responses to the viruses, with higher antibody titres in the resistant line [62–64]. MDV is a cell-associated herpesvirus and therefore strictly intracellular; however, both passively and actively acquired antibodies have been implicated in protective immunity against the disease [65]. Nevertheless, observing a strong antibody titre in field data could imply either enhanced resistance to pathogens or failure to launch an effective innate response. For example, a high SG titre may be indicative of an intracellular *Salmonella* carrier state (a feature of host-adapted *Salmonella*) or a recent infection [66]. Therefore, further studies are required to determine the desirable direction of selection on immune response that will enhance resistance and resilience to pathogens.

We confirmed three genomic regions that were previously shown to be associated with antibody response to different antigens [67, 68], one each on chromosomes 9 (for IBDV antibody titre), 18 (for IBDV and MDV antibody titre) and Z (for IBDV and PM antibody titre. The genomic region on chromosome 18 that was identified for MDV antibody titre is also consistent with previous studies on MDV survival of exotic birds [69]. In the genomic region for IBDV response on chromosome 9, two putative candidate genes, *protein tyrosine phosphatase non*-*receptor type 1* (*PTPN1*) and *nuclear factor of activated T*-*cells cytoplasmic calcineurin*-*dependent 2* (*NFATC2*), have been associated with efficient host response to viral infections in other species [70–72]. Moreover, the significant pathways and networks that we identified here for IBDV response are consistent with those identified in a whole-genome gene expression study of early responses to IBDV infection in inbred chicken lines with different resistance levels to IBDV [73]. We postulate that all these genomic regions may contribute to the underlying molecular mechanisms that are responsible for the overall effective host antibody response and hence segregate in many different chicken populations. However, further investigations are needed to identify the causative genes and elucidate the causative mutations in these regions.

### Selective sweeps

Analysis of selective sweeps revealed a positive signature of selection in the genomic region on chromosome 18 that harbours the *monocyte to macrophage differentiation*-*associated* (*MMD*) gene, which is involved in *Ras* signalling within the Golgi apparatus [74] and control of macrophage activation [75]. However, no direct association of this gene with antibody responses or parasitic disease resistance has been reported so far in the literature.

### Body weight and BCS

Our results for body weight and BCS are consistent with previous studies on exotic chicken [76–81] breeds and confirm many of the previously identified QTL. A recent GWAS of an F2 cross between Silky Fowl and White Plymouth Rock chickens also identified a genomic region that affects body weight on chromosome 4 [82], but it is located 6 Mb from the genomic region detected in our study. Moreover, the significant SNPs for BCS on chromosomes 8 and 15 that we detected are located in close proximity with the *LIM domain transcription factor* (*LMO4*) and the *LIM domain kinase 2* (*LIMK2*) genes, respectively. Gu et al. [82] also reported a strong association of the *LIM domain binding factor 2* (*LMO2*) locus with body weight.

### Genetic correlations between production, immune and disease traits

Taken together, the findings of our study support the view that simultaneous genetic improvement of enhanced antibody responses to bacterial and viral infections, increased resistance to major parasitic diseases, and that improved productivity is feasible in the two studied indigenous chicken ecotypes. There were no significant genetic correlations between immune, disease and production traits, which suggests that selection for enhanced immune response and resistance to parasitism would not compromise productivity. In addition, we found no SNPs that were significantly associated with production traits and, at the same time, also affected an immune or disease trait. Our results are consistent with field studies in sheep that reported neutral or even weakly favourable (negative) genetic correlations between gut parasitic infections and growth [18]. In addition, studies on turkeys [83] have shown that lines that are selected to grow more quickly may also have a stronger antibody response after challenge with an antigen from a pathogen compared to the slower growing lines. In all cases, multiple traits could be simultaneously included in the same breeding programme.

In the two chicken populations studied here, different heritabilities and different genomic regions may appear to control a given trait. These results support the hypothesis that indigenous chickens from distant geographic regions may have developed different adaptation mechanisms, which render them genetically distinct. Therefore, our findings support the current FAO recommendations that an appropriate and tailored response should be developed for each operating environment in the developing countries, as a “one-size-fits-all” approach is not usually successful for village poultry programmes [84]. Nevertheless, several common candidate genomic regions for body weight and disease resistance traits were also identified for the two ecotypes; which may indicate that a joint breeding programme comprising both ecotypes is worthy of further investigation.

## Conclusions

Based on our results, simultaneous genetic selection to enhance productivity, immune response, and health represents a valid possibility for the improvement of indigenous village chickens in Ethiopia and, by extension, other indigenous village chicken populations in sub-Saharan Africa and beyond. Such improvements will not only increase profitability but also animal welfare in areas where veterinary interventions are virtually not available. Technically, this may be achieved by selectively increasing the frequencies of target alleles and haplotypes within specific marker-assisted schemes. Genomic selection might be another option to improve the traits of interest; nevertheless, genotyping with the high-density SNP array used here would be prohibitively expensive to apply in the large-scale and might only be feasible to strategically genotype specific key individuals in a breeding programme. However, the construction of low-density custom SNP arrays based on the SNPs and regions identified in our study, as was effectively developed and applied in sheep [85], could provide practical cost-effective alternatives. Therefore, a custom-made array could be developed and used as an additional tool for genetic selection in non-phenotyped birds [85, 86] in the studied ecotypes. We also recommend that our results be validated with independent indigenous chicken ecotypes from other developing countries to corroborate their potential wider utility.

## Additional files

10.1186/s12711-016-0252-7 Descriptive statistics of all traits studied in Horro and Jarso chickens. Means and standard deviations (STD) for infectious bursal disease virus (IBDV), Mareks’ disease virus (MDV), Salmonella enterica serovar Gallinarum (SG) and Pasteurella multocida (PM) antibody titres; Eimeria and cestodes egg counts; body weight (kg) and body condition score (BCS, 0-3 scale) measurements.
10.1186/s12711-016-0252-7 Phenotypic and genetic correlations among all traits studied in Jarso chickens (Table S2) and Horro (Table S3) chickens. Phenotypic (above diagonal) and genetic correlation (below the diagonal) estimates (standard errors in parentheses) among the immune, disease and productivity traits.
10.1186/s12711-016-0252-7 Principal component analysis results for Horro (red triangles) and Jarso (blue squares) chickens.
10.1186/s12711-016-0252-7 Q–Q plots displaying the GWAS results for Jarso (Figure S2) and Horro (Figure S3) chickens. Description (Figure S2):Observed P-values are plotted against the expected P-values for (a) infectious bursal disease virus (IBDV) antibody titre, (b) Mareks’ disease virus (MDV) antibody titre, (c) Salmonella enterica serovar Galinarum (SG) antibody titre, (d) Pasteurella multocida (PM) antibody titre, (e) Eimeria parasitism resistance, (f) cestodes parasitism. Description (Figure S3): Observed P-values are plotted against the expected P-values for (a) infectious bursal disease virus (IBDV) antibody titre, (b) Mareks’ disease virus (MDV) antibody titre, (c) Salmonella enterica serovar Galinarum (SG) antibody titre, (d) Pasteurella multocida (PM) antibody titre, (e) cestodes parasitism resistance, (f) body condition score, (g) body weight.
10.1186/s12711-016-0252-7 Annotation of the significant SNPs identified from the GWAS for Jarso (Table S4) and Horro (Table S5) chickens. Linkage disequilibrium (r) among the SNPs identified within the same genomic region is also provided.
10.1186/s12711-016-0252-7 List of genes located in the candidate genomic regions for the Jarso (Table S6) and Horro (Table S7) chickens. Candidate regions were defined 100 kb upstream and downstream of the significant SNPs. Gene lists for infectious bursal disease virus (IBDV) antibody titre, Mareks’ disease virus (MDV) antibody titre, Salmonella enterica serovar Galinarum (SG) antibody titre, Pasteurella multocida (PM) antibody titre, Eimeria parasitism resistance, cestodes parasitism resistance, body weight and body condition score (BCS) are shown.
10.1186/s12711-016-0252-7 Pathway analysis results using the IPA software for the Jarso (Figure S4) and Horro (Figure S5) chickens. Description (Figure S4): The most highly represented canonical pathways for the genes located in the candidate genomic regions for (a) infectious bursal disease virus (IBDV) antibody titre, (b) Mareks’ disease virus (MDV) antibody titre, (c) Pasteurella multocida (PM) antibody titre, (d) Eimeria parasitism resistance, (e) cestodes parasitism resistance,(f) body weight, (g) body condition score (BCS). The solid yellow line represents the significance threshold. The line with squares represents the ratio of the genes within each pathway to the total number of genes in the pathway. Description (Figure S5): The most highly represented canonical pathways for the genes located in the candidate genomic regions for (a) infectious bursal disease virus (IBDV) antibody titre, (b) Mareks’ disease virus (MDV) antibody titre, (c) Salmonella enterica serovar Galinarum (SG) antibody titre, (d) Pasteurella multocida (PM) antibody titre (e) cestodes parasitism resistance, (f) body weight. The solid yellow line represents the significance threshold. The line with squares represents the ratio of the genes within each pathway to the total number of genes in the pathway.
10.1186/s12711-016-0252-7 Network analysis results using IPA software for the Jarso (Figure S6) and Horro (Figure S7) chickens. Description (Figure S6): The networks illustrate the molecular interactions between products of genes selected from candidate genomic regions for infectious bursal disease virus (IBDV) antibody titre (networks A and B); Mareks’ disease virus (MDV) antibody titre (C); Eimeria parasitism resistance (E and F); cestodes parasitism resistance (G and H); body condition score (BCS, I). Solid and dashed arrows represent direct and indirect interactions, respectively. The white colour indicates gene products added to the IPA analysis because of their interaction with the target gene products. Description (Figure S7): The networks illustrate the molecular interactions between products of genes selected from the candidate genomic regions for Salmonella enterica serovar Galinarum (SG) antibody titre (network A); for Pasteurela multocida (PM) antibody titre (B); for cestodes parasitism resistance (C , D and E); for body weight (F). Solid and dashed arrows represent direct and indirect interactions, respectively. The white colour indicates gene products added to the IPA analysis because of their interaction with the target gene products.
10.1186/s12711-016-0252-7 Functional annotation clustering analysis using DAVID software for Jarso and Horro chickens. The most highly represented functional gene clusters located in the candidate genomic regions for infectious bursal disease virus (IBDV) antibody titre, Salmonella enterica serovar Galinarum (SG) antibody titre, Pasteurella multosida (PM) antibody titre, Eimeria parasitism resistance, cestodes parasitism resistance and body condition score (BCS).
10.1186/s12711-016-0252-7 List of selected candidate genes for immune, disease and productivity traits studied in Jarso (Table S9) and Horro (Table S10) chickens.
