# Supplementary material for: Genome-wide association studies of immune, disease and production traits in indigenous chicken ecotypes
Source: Genet Sel Evol. 2016 Sep 29;48:74. doi: 10.1186/s12711-016-0252-7 (PMC5041578; doi:10.1186/s12711-016-0252-7)
Supplement: Supplementary file 1 — 10.1186/s12711-016-0252-7 Descriptive statistics of all traits studied in Horro and Jarso chickens. Means and standard deviations (STD) for infectious bursal disease virus (IBDV), Mareks’ disease virus (MDV), Salmonella enterica serovar Gallinarum (SG) and Pasteurella multocida (PM) antibody titres; Eimeria and cestodes egg counts; body weight (kg) and body condition score (BCS, 0-3 scale) measurements. [file 12711_2016_252_MOESM1_ESM.docx]

**Additional File 1: Table S1. Descriptive statistics of all traits studied** **in Horro and Jarso chickens.** Means and standard deviations (STD) for Infectious Bursal Disease Virus (IBDV), Mareks’ disease virus (MDV), *Salmonella enterica* serovar Gallinarum (SG) and *Pasteurella multocida* (PM) antibody titres; *Eimeria* and *cestodes* egg counts; body weight (kg) and body condition score (BCS, 0-3 scale) measurements.

| **Trait** | | **Horro** | | | **Jarso** | |
| --- | --- | --- | --- | --- | --- | --- |
|  | **Mean** | | **STD** | **Mean** | | **STD** |
| IBDV | 0.063 | | 0.175 | 0.009 | | 0.123 |
| MDV | 0.190 | | 0.607 | 0.238 | | 0.561 |
| SG | 1.430 | | 1.830 | 1.190 | | 0.970 |
| PM | 1.024 | | 0.999 | 0.887 | | 0.537 |
| *Eimeria* | 27.900 | | 118.300 | 50.000 | | 287.800 |
| Cestodes | 1.280 | | 5.940 | 4.570 | | 37.540 |
| Body Weight | 1.390 | | 0.340 | 1.260 | | 0.280 |
| BCS | 1.600 | | 0.600 | 1.500 | | 0.600 |

**IBDV, MDV, SG, PM antibody titres measured as ELISA s:p ratio = (mean sample OD-negative control OD)/ (positive control OD- negative control OD); *Eimeria* and cestodes parasitism measured as egg counts/g of feacal**
