# Supplementary material for: Genome-wide association studies of immune, disease and production traits in indigenous chicken ecotypes
Source: Genet Sel Evol. 2016 Sep 29;48:74. doi: 10.1186/s12711-016-0252-7 (PMC5041578; doi:10.1186/s12711-016-0252-7)
Supplement: Supplementary file 2 — 10.1186/s12711-016-0252-7 Phenotypic and genetic correlations among all traits studied in Jarso chickens (Table S2) and Horro (Table S3) chickens. Phenotypic (above diagonal) and genetic correlation (below the diagonal) estimates (standard errors in parentheses) among the immune, disease and productivity traits. [file 12711_2016_252_MOESM2_ESM.docx]

**Additional File 2: Table S2** **and Table S3**

**Table S2. Phenotypic and genetic correlations among all traits studied in Jarso. chickens.** Phenotypic (above diagonal) and genetic correlation (below diagonal) estimates (standard errors in parentheses) among the immune, disease and productivity traits.

| Trait | IBDV | MDV | SG | PM | *Eimeria* | Cestodes | Body  weight | BCS |
| --- | --- | --- | --- | --- | --- | --- | --- | --- |
| IBDV |  | 0.02  (0.05) | 0.05  (0.05) | 0.08  (0.05) | 0.07  (0.05) | 0.05  (0.05) | 0.03  (0.06) | 0.07  (0.05) |
| MDV | -0.24  (0.28) |  | **-0.14**  **(0.05)** | -0.03  (0.05) | 0.03  (0.05) | -0.04  (0.05) | -0.03  (0.06) | -0.01  (0.05) |
| SG | **0.94**  **(0.46)** | 0.35  (0.39) |  | **0.41**  **(0.04)** | **-0.11**  **(0.05)** | -0.006  (0.05) | 0.09  (0.05) | 0.02  (0.05) |
| PM | 0.08  (0.53) | -0.43  (0.42) | **0.98**  **(0.16)** |  | -0.05  (0.05) | **-0.10**  **(0.05)** | **0.10**  **(0.05)** | 0.04  (0.05) |
| *Eimeria* | 0.15  (0.35) | -0.16  (0.25) | -0.02  (0.41) | -0.42  (0.41**)** |  | **0.19**  **(0.05)** | -0.01  (0.05) | **0.11**  **(0.05)** |
| Cestodes | -0.06  (0.39) | 0.07  (0.28) | 0.34  (0.47) | 0.84  (0.51) | **0.68**  **(0.25)** |  | 0.09  (0.06) | 0.04  (0.05) |
| Body weight | 0.06  (0.28) | -0.03  (0.21) | 0.17  (0.36) | 0.18  (0.33) | -0.002  (0.23) | 0.04  (0.26) |  | **0.40**  **(0.04)** |
| BCS | 0.26  (0.31) | 0.28  (0.23) | 0.12  (0.45) | -0.05  (0.44) | 0.36  (0.27) | 0.10  (0.31) | **0.50**  **(0.20)** |  |
| Estimates in bold attained statistical significance (P<0.05). IBDV: antibody titres to Infectious bursal disease virus; MDV: antibody titres to Mareks’ disease virus; SG: antibody titres to *Salmonella enterica* serovar Gallinarum; PM: antibody titres to *Pasteurella multocida*; BCS : body condition score. | | | | | | | | |

**Table S3. Phenotypic and genetic correlations among all traits studied in Horro chickens.** Phenotypic (above diagonal) and genetic correlation (below the diagonal) estimates (standard errors in parentheses) among the immune, disease and productivity traits.

| Trait | IBDV | MDV | SG | PM | *Eimeria* | Cestodes | Body  weight | BCS |
| --- | --- | --- | --- | --- | --- | --- | --- | --- |
| IBDV |  | 0.05  (0.05) | **0.22**  **(0.05)** | **0.10**  **(0.05)** | **-0.11**  **(0.05)** | 0.02  (0.05) | **0.32**  **(0.06)** | 0.04  (0.05) |
| MDV | 0.26  (0.20) |  | -0.01  (0.55) | 0.007  (0.05) | 0.05  (0.05) | -0.01  (0.05) | 0.04  (0.05) | 0.07  (0.05) |
| SG | **0.53**  **(0.26)** | -0.01  (0.02) |  | **0.41**  **(0.04)** | **-0.10**  **(0.05)** | -0.004  (0.05) | **0.16**  **(0.05)** | -0.02  (0.05) |
| PM | 0.22  (0.20) | 0.45  (0.31) | **0.97**  **(0.46)** |  | **-0.16**  **(0.05)** | **-0.10**  **(0.05)** | **0.10**  **(0.05)** | 0.09  (0.05) |
| *Eimeria* | 0.00  (0.00) | 0.00  (0.00) | 0.00  (0.00) | 0.00  (0.00) |  | **0.12**  **(0.05)** | -0.013  (0.05) | -0.05  (0.05) |
| Cestodes | -0.17  (0.29) | 0.64  (0.44) | 0.92  (0.95) | -0.24  (0.40) | **-**0.00  (3.28) |  | **-**0.006  (0.05) | -0.02  (0.05) |
| Body weight | 0.28  (0.15) | 0.21  (0.26) | 0.58  (0.67) | -0.10  (0.29) | 0.00  (0.00) | 0.30  (0.47) |  | **0.42**  **(0.04)** |
| BCS | -0.38  (0.42) | -0.98  (0.65) | -0.97  (0.75) | 0.97  (0.62) | 0.00  (0.00) | -0.71  (0.39) | **0.97**  **(0.31)** |  |
| Estimates in bold attained statistical significance (P<0.05). IBDV: antibody titres to Infectious bursal disease virus; MDV: antibody titres to Mareks’ disease virus; SG: antibody titres to *Salmonella enterica* serovar Gallinarum; PM: antibody titres to *Pasteurella multocida*; BCS ~ body condition score. | | | | | | | | |
