# Supplementary figures and images for: Genome-wide association studies of immune, disease and production traits in indigenous chicken ecotypes
Source: Genet Sel Evol. 2016 Sep 29;48:74. doi: 10.1186/s12711-016-0252-7 (PMC5041578; doi:10.1186/s12711-016-0252-7)

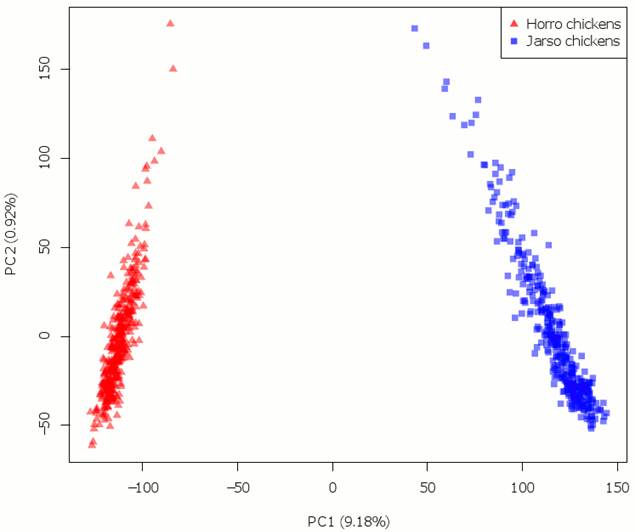

Supplement: Supplementary file 3 — 10.1186/s12711-016-0252-7 Principal component analysis results for Horro (red triangles) and Jarso (blue squares) chickens. [file 12711_2016_252_MOESM3_ESM.jpg]
