# Supplementary material for: Genome-wide association studies of immune, disease and production traits in indigenous chicken ecotypes
Source: Genet Sel Evol. 2016 Sep 29;48:74. doi: 10.1186/s12711-016-0252-7 (PMC5041578; doi:10.1186/s12711-016-0252-7)
Supplement: Supplementary file 4 — 10.1186/s12711-016-0252-7 Q–Q plots displaying the GWAS results for Jarso (Figure S2) and Horro (Figure S3) chickens. Description (Figure S2):Observed P-values are plotted against the expected P-values for (a) infectious bursal disease virus (IBDV) antibody titre, (b) Mareks’ disease virus (MDV) antibody titre, (c) Salmonella enterica serovar Galinarum (SG) antibody titre, (d) Pasteurella multocida (PM) antibody titre, (e) Eimeria parasitism resistance, (f) cestodes parasitism. Description (Figure S3): Observed P-values are plotted against the expected P-values for (a) infectious bursal disease virus (IBDV) antibody titre, (b) Mareks’ disease virus (MDV) antibody titre, (c) Salmonella enterica serovar Galinarum (SG) antibody titre, (d) Pasteurella multocida (PM) antibody titre, (e) cestodes parasitism resistance, (f) body condition score, (g) body weight. [file 12711_2016_252_MOESM4_ESM.docx]

**Additional File 5: Figure S2 and** **Figure S3.**

**Figure S2. Q–Q plots displaying the GWAS results for Jarso chickens.** Observed P-values are plotted against the expected P-values for **a)** Infectious bursal disease virus (IBDV) antibody titre, **b)** Mareks’ disease virus (MDV) antibody titre, **c)** *Salmonella enterica* serovan Galinarum (SG) antibody titre, **d)** *Pasteurella multocida* (PM) antibody titre, **e)** *Eimeria* parasitism resistance, **f)** cestodes parasitism resistance, **g)** body condition score (BCS), **h)** body weight.

1. **IBDV antibody titre**

**
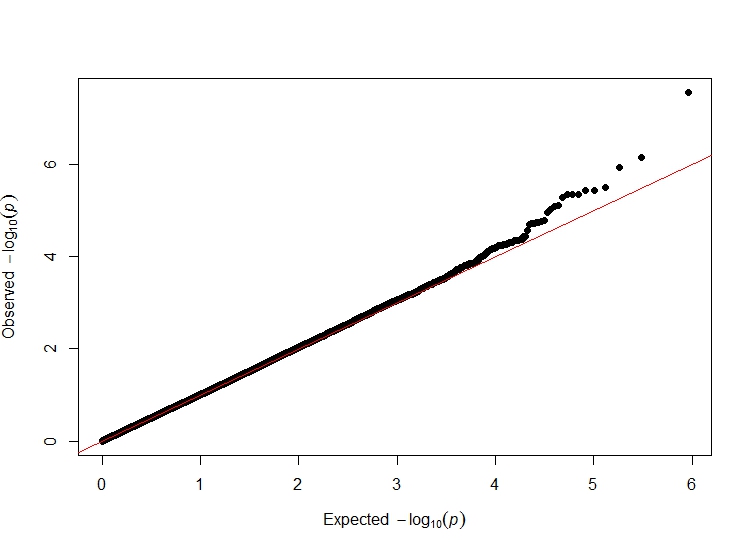
**

1. **MDV antibody titre**

**
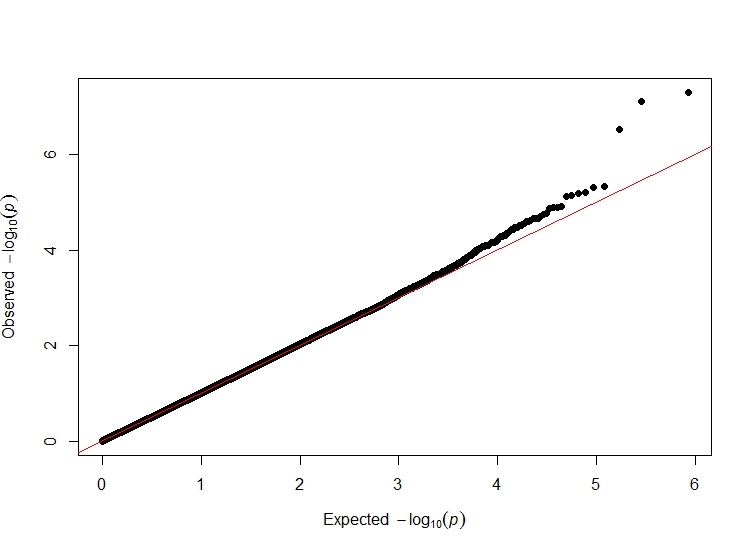
**

1. **SG antibody titre**

**
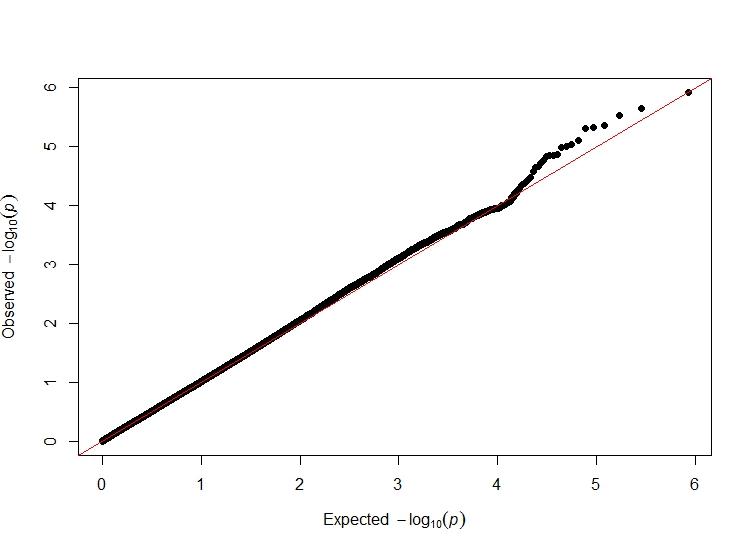
**

1. **PM antibody titre**

**
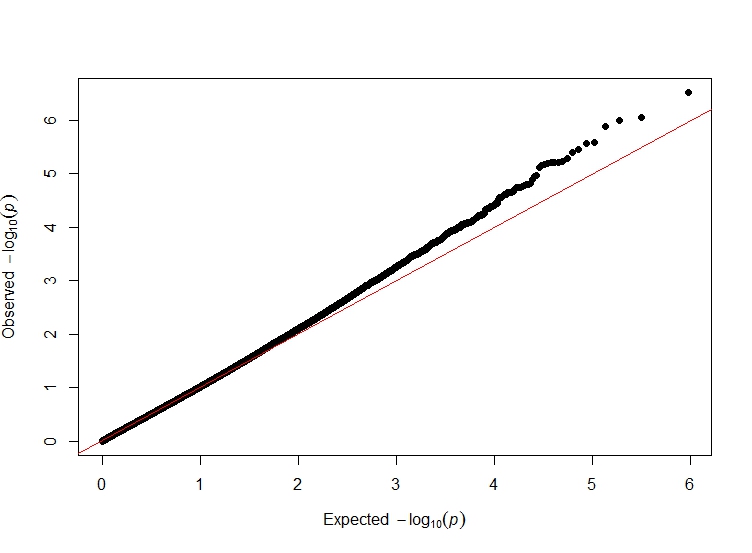
**

1. ***Eimeria* parasitism resistance**

**
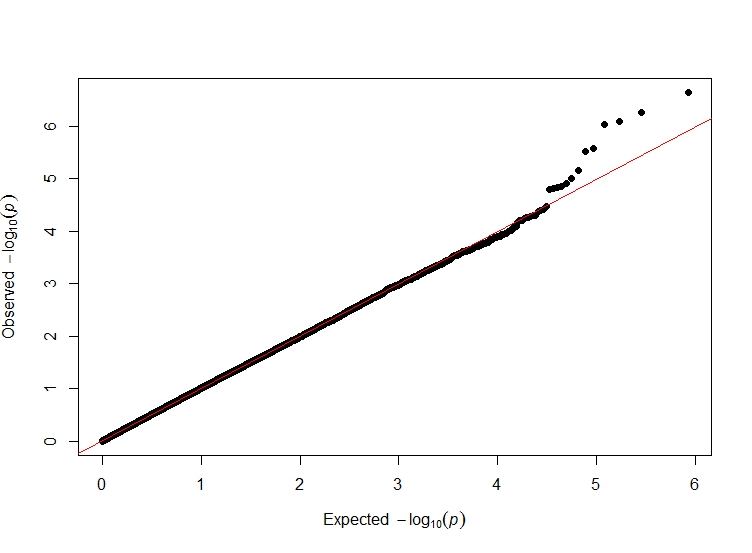
**

1. **Cestodes parasitism resistance**

**
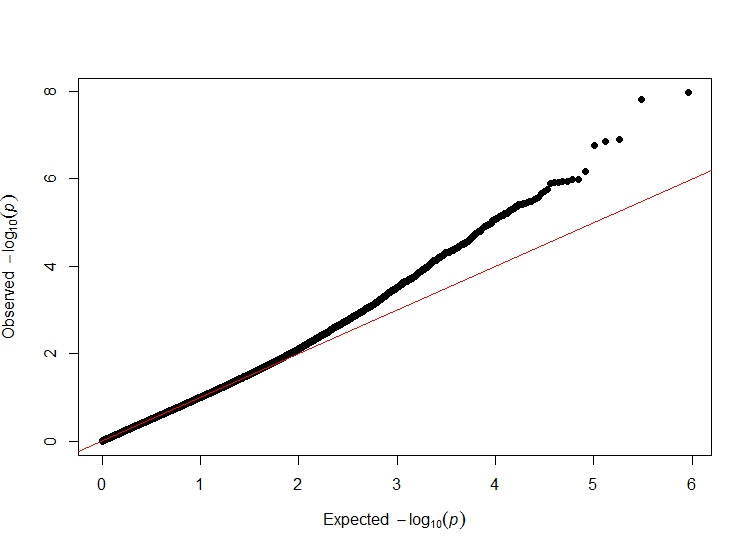
**

1. **Body condition score**

**
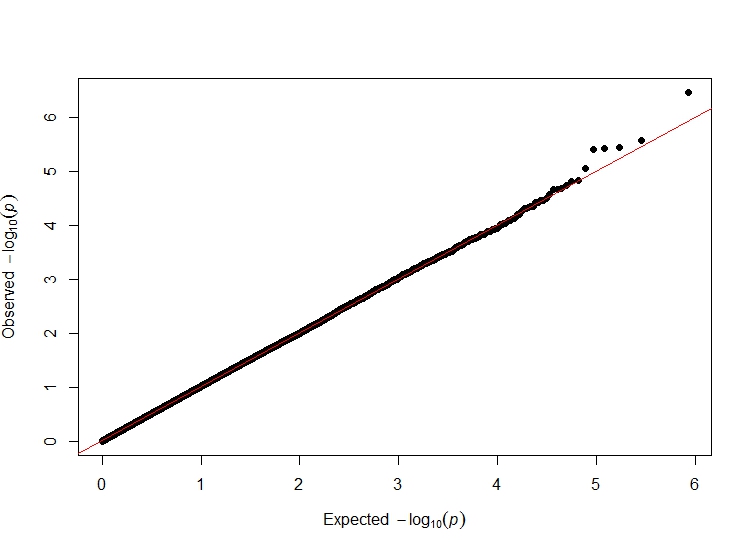
**

1. **Body weight**

**
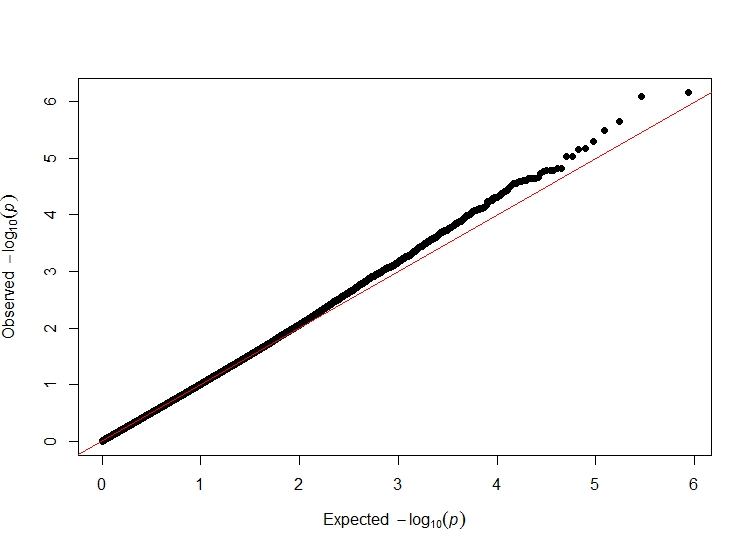
**

**Figure S3.** **Q–Q plots displaying the GWAS results for Horro chickens.** Observed P-values are plotted against the expected P-values for **a)** Infectious bursal disease virus (IBDV) antibody titre, **b)** Mareks’ disease virus (MDV) antibody titre, **c)** *Salmonella enterica* serovar Galinarum (SG) antibody titre, d) *Pasteurella multocida* (PM) antibody titre, **e)** cestodes parasitism resistance, **f)** body condition score, **g)** body weight.

1. **IBDV antibody titre
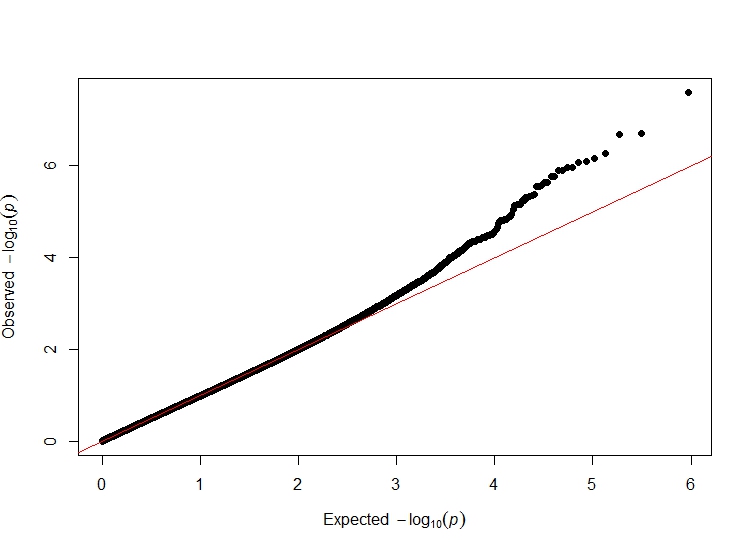
**

**b) MDV antibody titre
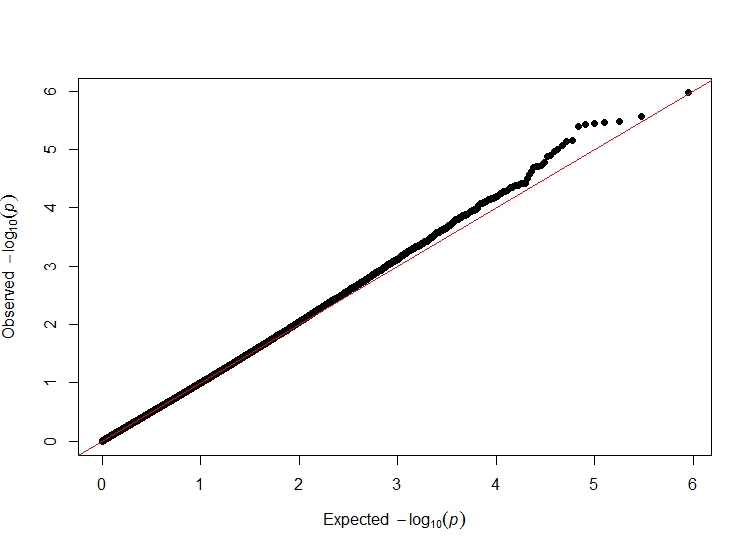
**

1. **SG antibody titre**

**
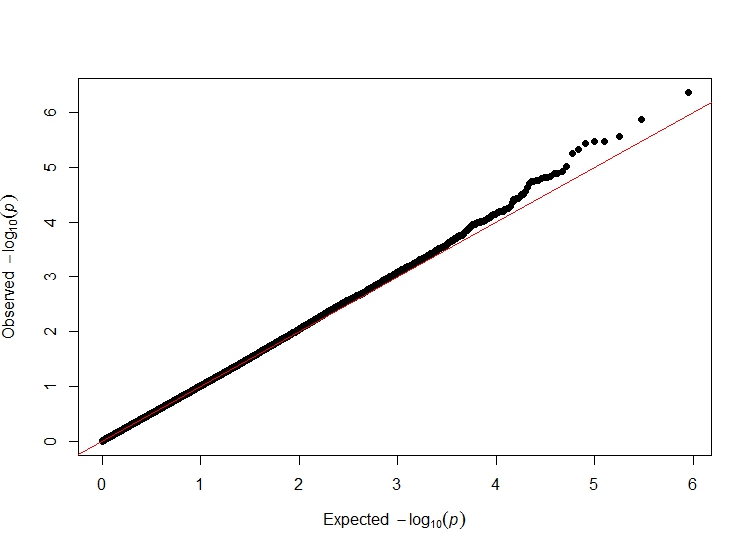
**

1. **PM antibody titres**

**
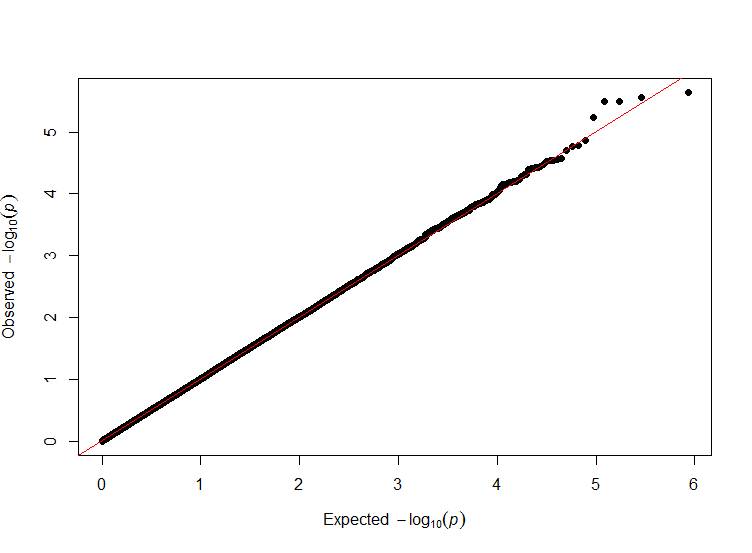
**

1. **Cestodes parasitism resistance**

**
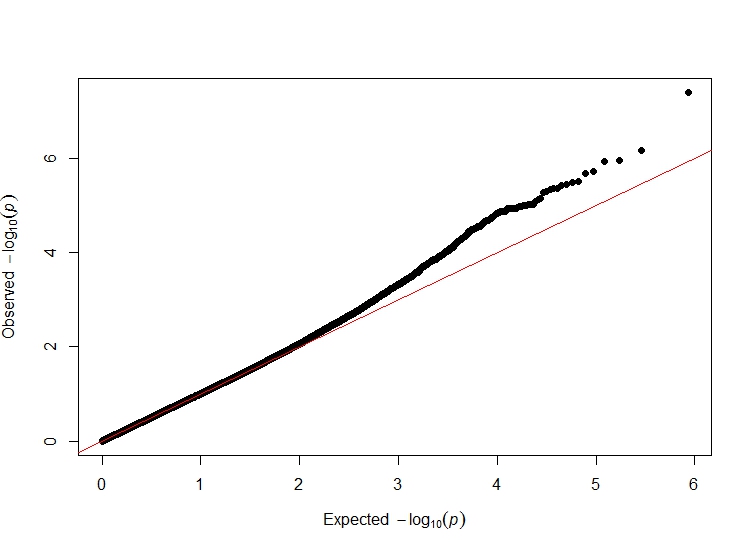
**

1. **Body condition score**

**
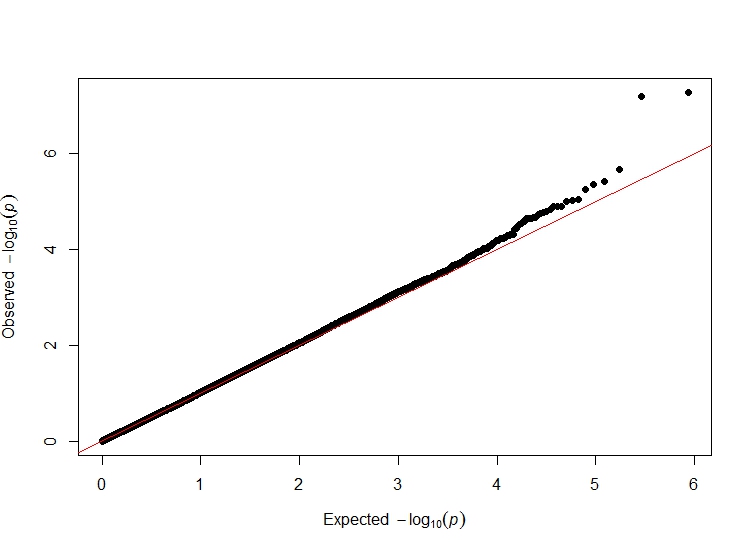
**

1. **Body weight**

**
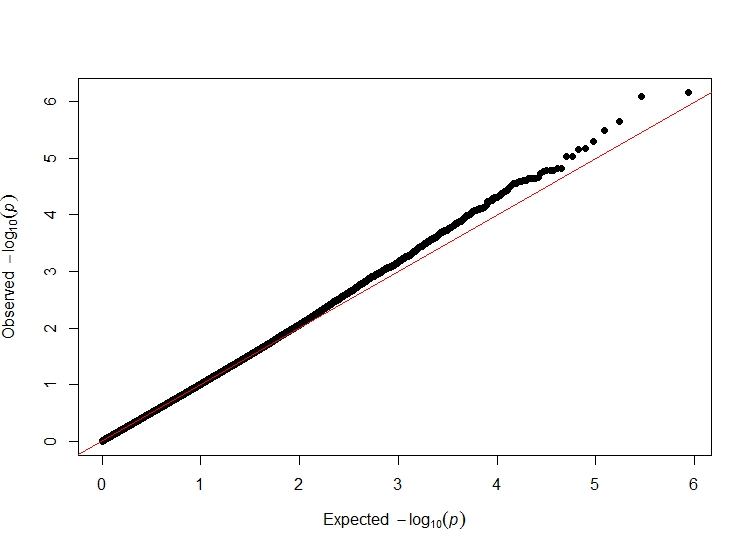
**
