# Supplementary material for: Genome-wide association studies of immune, disease and production traits in indigenous chicken ecotypes
Source: Genet Sel Evol. 2016 Sep 29;48:74. doi: 10.1186/s12711-016-0252-7 (PMC5041578; doi:10.1186/s12711-016-0252-7)
Supplement: Supplementary file 5 — 10.1186/s12711-016-0252-7 Annotation of the significant SNPs identified from the GWAS for Jarso (Table S4) and Horro (Table S5) chickens. Linkage disequilibrium (r) among the SNPs identified within the same genomic region is also provided. [file 12711_2016_252_MOESM5_ESM.docx]

**Additional File 5: Table S4 and Table S5.**

**Table S4. Annotation of the significant SNPs identified from GWAS for Jarso chickens.** Linkage disequilibrium (r^2^) among the SNPs identified within the same genomic region is also provided.

| **Trait** | **SNP** | **Location**  **Chr (bp)** | **Alleles**  **Ref/SNP** | **Annotation** |
| --- | --- | --- | --- | --- |
| IBDV | Affx-51021753 | 20(13729523) | A/G | Synonymous variant for *MOCS3* protein,  upstream gene variant to dolichyl-phosphate mannosyltransferase polypeptide 1, catalytic subunit(*DPM1*) |
| IBDV | Affx-50919107 | 2(40486311) | A/G | intron variant in glutamate decarboxylase-like 1 (*GADL1*) |
| IBDV | Affx-51897552 | Z (2805087) | C/T | Intron variant in set binding protein 1 (*SETBP1*) |
| IBDV | Affx-51897506 | Z (28519476) | C/T | Intergenic variant between protein tyrosine phosphatase, receptor type, D (*PTPRD*) and an uncharacterised protein |
|  | Affx-50554286 | 11(8097578) | A/G | Intergenic variant between prefoldin-like chaperone (*URI1*) and  zinc finger protein 536 (*Zfp536*) |
| MDV  r^2^=0.3-0.4 | Affx-50554294 | 11(8102461) | A/G | Intergenic variant between prefoldin-like chaperone (*URI1*) and  zinc finger protein 536 (*Zfp536*) |
|  | Affx-50554208 | 11(8066972) | C/T | Intergenic variant between prefoldin-like chaperone (*URI1*) and  zinc finger protein 536 (*Zfp536*) |
| MDV | Affx-50613892 | 13(14006092) | A/G | Intron variant in sparc/osteonectin, cwcv and kazal-like domains proteoglycan 1 (*SPOCK1*) |
| SG  r^2^=0.95 | Affx-50739265 | 17(9157484) | C/T | Intron variant in DENN/MADD domain containing 1A (*DENND1A*) |
|  | Affx-50739250 | 17(9151381) | T/G | Upstream_gene_variant uc_338 |
| SG | Affx-51614950 | 5(6690649) | G/T | Intergenic variant between synaptotagmin IX (*SYT9*) and olfactomedin-like 1 |
| PM | Affx-50315740 | 1(194369733) | C/A | Intron variant in FCH and double SH3 domains 2 (*FCHSD2*) |
| PM | Affx-51210522 | 3(13285408) | A/C | Intron variant in Gallus gallus p21 protein (Cdc42/Rac)-activated kinase 7 (*PAK7*) |
| PM | Affx-50856253 | 2(135976613) | C/T | Intron variant in Collectin-10 (*COLEC10*) |
| PM | Affx-51091424 | 23(3294718 ) | C/T | Intergenic variant between ras-related GTP-binding protein C and  UTP11-like, U3 small nucleolar ribonucleoprotein |
| PM | Affx-51891828 | Z (22361089) | C/T | Upsteram gene variant arylsulfatase B  Downstream gene variant dimethylglycine dehydrogenase |
| *Eimeria*  r^2^=0.61 | Affx-51295947 | 3(60276817) | G/T | Intergenic variant between Na+/K+ transporting ATPase interacting 2 and triadin |
|  | Affx-51295976 | 3(60299318) | G/T | Intergenic variant between Na+/K+ transporting ATPase interacting 2 and triadin |
| *Eimeria*  r^2^=0.88 | Affx-50757437 | 18(5763355) | T/C | Intergenic variant between carbonic anhydrase X and  target of myb1 like 1 membrane trafficking protein |
|  | Affx-50757438 | 18(5763659) | T/G | Intergenic variant between carbonic anhydrase X and  target of myb1 like 1 membrane trafficking protein |
| *Eimeria* | Affx-51550767 | 5(28051272) | C/T | Intergenic between Transmembrane protein 229B and  pleckstrin homology domain containing, family H (with MyTH4 domain) member 1 |
| *Eimeria* | Affx-50711670 | 16(146715) | G/T | Intron variant *TRIM27.2*  Upstream gene variant *TRIM27.1*  Downstream gene variant *GNB2L1*  Downstream gene variant *SNORD95* |
| Cestodes | Affx-51718143 | 7(21664924) | G/C | Intron variant aspartyl aminopeptidase (*DNPEP*)  Intron variant SPEG complex locus (*SPEG*) |
| Cestodes | Affx-50667122 | 14(5803456) | T/C | Intron variant lipase maturation factor 1(*LMF1*) |
| Cestodes | Affx-50796263 | 19(9284997) | A/C | Intron variant Nemo-like kinase (*NLK*) |
| Cestodes | Affx-50805630 | 2(104948558) | A/C | Intergenic variant between cadherin 2 and desmocollin |
| Cestodes | Affx-50417651 | 1(7326327) | C/T | 3 prime UTR variant Histone-lysine N-methyltransferase SUV39H2  3 prime UTR variant protein artemis DCLRE1C (reverse strand) |
| Cestodes | Affx-50350190 | 1(37252069) | G/T | Intergenic between thyrotropin-releasing hormone degrading enzyme and potassium channel, voltage gated Shaw related subfamily C, member 2 |
|  | Affx-51792456 | 8(22438049) | C/T | Intergenic variant between BEN domain containing 5 and ELAV-like protein 4 |
| Cestodes  r^2^=0.6-0.8 | Affx-51792501 | 8(22456586) | G/A | Intergenic variant between BEN domain containing 5 and ELAV-like protein 4 |
|  | Affx-51792503 | 8(22457826) | T/G | Intergenic variant between BEN domain containing 5 and ELAV-like protein 4 |
|  |  |  |  |  |
| Cestodes | Affx-51875683 | 9(8596899) | A/G | Intergenic variant neuronal tyrosine-phosphorylated phosphoinositide-3-kinase  adaptor 2 |
| Cestodes | Affx-51474665 | 4(72218113) | C/T | Intergenic variant between protocadherin 7 and stromal interaction molecule 2 |
| Cestodes | Affx-51675956 | 6(34224060) | A/G | Intergenic variant between transcription elongation regulator 1-like  and serine/threonine-protein phosphatase 2A 55 kDa regulatory subunit B delta isoform |
| Cestodes | Affx-51894886 | Z(24758394) | A/T | Downstream gene variant dimethylglycine dehydrogenase |
| Cestodes | Affx-50712683 | 16(80940) |  | Intron variant in an uncharacterised protein orthologue to major histocompatibility complex, class II, DM beta (*HLA-DMB*) (in humans)  Upstream variant in Major histocompatibility complex class II beta chain BMA2, (similar to HLA class II, D beta chain) precursor (*DMB2*)  Downstream variant in B locus M alpha chain 1 precursor (*DMA*), and downstream variant in antigen peptide transporter 1(*TAP1*) |
| Body weight  r^2^=0.2-1 | Affx-51502208 | 4(87162290) | A/G | All the SNP correspond to intergenic region variants between Gallus gallus catenin (cadherin-associated protein) alpha 2 (*CTNNA2*) and an uncharacterised protein; and they are also  downstream gene variants from a novel microRNA |
|  | Affx-51502179 | 4(87149557) | A/G |  |
|  | Affx-51502172 | 4(87146841) | G/A |  |
|  | Affx-51502191 | 4(87154473) | C/T |  |
|  | Affx-51502955 | 4(87546243) | C/T |  |
|  | Affx-51502405 | 4(87266160) | T/C |  |
|  | Affx-51501548 | 4(86818215) | C/T |  |
|  | Affx-51502756 | 4(87449896) | C/A |  |
|  | Affx-51502383 | 4(87254896) | A/C |  |
|  | Affx-51501235 | 4(86663849) | T/C |  |
|  | Affx-51502311 | 4(87215318) | G/A |  |
|  | Affx-51501231 | 4(86662441) | C/T |  |
|  | Affx-51502367 | 4(87244290) | A/C |  |
|  | Affx-51502246 | 4(87182268) | C/T |  |
|  | Affx-51500484 | 4(86290949) | A/C |  |
|  | Affx-51502298 | 4(87208267) | A/G |  |
|  | Affx-51502304 | 4(87211838) | A/G |  |
|  | Affx-51501571 | 4(86830246) | T/C |  |
| Body weight  r^2^=0.49 | Affx-51501414 | 4(784066) | T/C | Intergenic variant between two undescribed proteins ortholoques of relaxin and insulin-like family peptide receptor 2, like, respectively |
|  | Affx-51501208 | 4(783086) | T/G | Intergenic variant between two undescribed proteins ortholoques of relaxin and insulin-like family peptide receptor 2, like, respectively |
| BCS | Affx-50734945 | 17(7814709) | A/G | Intron variant in notch1 |
| BCS | Affx-51773927 | 8(14332889) | C/A | Intergenic variant between  protein kinase N2 and LIM domain transcription factor (*LMO4*) |
| BCS | Affx-51160997 | 27( 3533019) | A/G | Downstream gene variant homeobox B13 (*HOX13*) |
| IBDV: antibody titres to Infectious bursal disease virus; MDV: antibody titres to Mareks’ disease virus; SG: antibody titres to *Salmonella enterica* serovan Gallinarum; PM: antibody titres to *Pasteurella multocida*; BCS : body condition score. Annotation was performed based on galgal4 assembly. Linkage disequilibrium (LD) as r^2^ estimates are also presented for SNPs in LD. | | | | |

**Table S5.** A**nnotation of the significant SNPs identified from the GWAS for Horro chickens.** Linkage disequilibrium (as r^2^ estimation) among the SNPs identified within the same genomic region is also provided.

| **Trait** | **SNP** | **Location**  **Chr (bp)** | **Alleles**  **Ref/SNP** | **Annotation** |
| --- | --- | --- | --- | --- |
| IBDV | Affx-51526157 | 5(15315358) | C/T | Intron variant in leucine rich repeat containing 56 gene (*LRC56*) |
| IBDV | Affx-51242536 | 3(3148207) | C/T | Intron variant in tyrosine-protein kinase Mer precursor (*MERTK*) |
| IBDV | Affx-50862142 | 2(139341263) | T/G | Intergenic variant between the gene Myc proto-oncogene protein  and a novel micro RNA |
| IBDV | Affx-51878048 | 9(866678) | T/C | Synonymous variant in myosin VIIB gene (*MYO7B* ) |
| IBDV | Affx-51183095 | 28(581149) | T/C | Intron variant in signal peptide peptidase-like 2B precursor gene (*SPPL2B*) |
| IBDV | Affx-51884018 | Z(15058127) | T/C | Intron variant in Insulin gene enhancer protein ISL-1 |
| IBDV | Affx-50756295 | 18(5404597) | T/C | Intergenic variant between carbonic anhydrase X and  target of myb1 like 1 membrane trafficking protein |
| IBDV | Affx-51084536 | 23(1467133) | A/C | Missense variant (deleterious, aminoacids W/C) in XK-related protein 8 (*XKR8*)  Downstream gene variant to EYA transcriptional coactivator and phosphatase 3 (*EYA3*)  Downstream gene variant to acid sphingomyelinase-like phosphodiesterase 3b precursor (*SMPDL3B*) |
| IBDV | Affx-50584797 | 12(19824359) | G/T | Intron variant in FYVE, RhoGEF and PH domain containing 5 gene (*FGD5*) |
| MDV | Affx-51262165 | 3(42096244) | T/C | Intron variant in ribosomal protein S6 kinase, 90kDa, polypeptide 2 (*RPS6KA2*) |
| MDV | Affx-50589622 | 12(3932659) | A/C | Intron variant ATPase, Ca++ transporting, plasma membrane 2 (*ATP2B2*) |
| MDV | Affx-50758514 | 18(6099330) | T/C | Intron variant in ankyrin-repeat and fibronectin type III domain containing 1 |
| SG | Affx-50376191 | 1(51661206) | A/C | Missense variant (deleterious, aminoacids K/N) in myosin-9 gene (*MYH9*) |
| SG | Affx-51254552 | 3(38112387) | C/T | Intergenic variant between two uncharacterised proteins |
| SG | Affx-50583084 | 12(19159478) | C/A | Intergenic variant between LIM and cysteine-rich domains 1 and caveolin-3 (*CAV-3*) |
| SG | Affx-50712674 | 16(78709) |  | Iintron variant Major histocompatibility complex class II beta chain *BMA2*, (similar to HLA class II, D beta chain) precursor (*DMB2*)  Upstrean variant MHC class I alpha chain 1 (*BF1*)  Downstream variant antigen peptide transporter 1 (*TAP1*), B locus M alpha chain 1 precursor (*BMA*), and an uncharacterised protein orthologue to *HLA-DMB* (in humans) |
| Cestodes | Affx-51266852 | 3(44583022) | A/C | Missense variant (aminoacids H/Q) in  mitogen-activated protein kinase kinase kinase 4 gene (*MAP3K4*) |
| Cestodes | Affx-50313244 | 1(193416334) | T/C | Intergenic variant between nuclear mitotic apparatus protein 1  and leucine carboxyl methyltransferase 2 |
|  |  | 1(18927296) |  | Intergenic variant between Pim-3 proto-oncogene, serine/threonine kinase and interleukin 17 receptor E-like |
| Cestodes | Affx-51085176 | 23(1631142) | T/C | Intergenic variant between heterogeneous nuclear ribonucleoprotein R  and leucine zipper protein 1 |
| Body weight | Affx-51856375 | 9(22119113) | T/C | Intergenic variant between short stature homeobox 2 and  PQ loop repeat containing 2-like |
| Body weight | Affx-50919051 | 2(40447782) | C/T | Downstream gene variant (208bp) TGF-beta receptor type-2 (*TGFBR2*) |
| Body weight | Affx-51500100 | 4(86100031) | G/A | Intron variant in an uncharacterised protein |
| Body weight | Affx-50595206 | 12(6188503) | G/A | Intergenic variant between homeobox protein BarH-like 1b and  PHD finger protein 2 |
|  | Affx-51794123 | 8(23157421) | T/C | Intron variant in Thioredoxin Domain Containing 12 (*TXNDC12*),  Upstream gene variant to Basic Transcription Factor 3-Like 4 (*BTF3L4*) |
| BCS  r^2^=0.8-1 | Affx-51794141 | 8(23164238) | C/T | Synonymous variant in *BTF3L4*, upstream gene variant *TXNDC12* |
|  | Affx-51794116 | 8(23154277) | C/T | Intron variant *TXNDC12* |
| BCS | Affx-50709156 | 15(8941550) | T/C | Upstream gene variant to Phosphatidylserine  Decarboxylase gene (*PISD*) |
| IBDV: antibody titres to Infectious bursal disease virus; MDV: antibody titres to Mareks’ disease virus; SG: antibody titres to *Salmonella enterica* serovan Gallinarum; BCS : body condition score. Annotation was performed based on galgal4 assembly. Linkage disequilibrium (LD) as r^2^ estimates are also presented for SNPs in LD. | | | | |
