# Supplementary material for: Genome-wide association studies of immune, disease and production traits in indigenous chicken ecotypes
Source: Genet Sel Evol. 2016 Sep 29;48:74. doi: 10.1186/s12711-016-0252-7 (PMC5041578; doi:10.1186/s12711-016-0252-7)

**Additional File 7: Figure S4 and Figure S5.**

**Figure S4. Pathway analysis results using the IPA software for the Jarso chickens**. The most highly represented canonical pathways of genes located at the candidate genomic regions for a) Infectious bursal disease virus (IBDV) antibody titre, b) Mareks’ disease virus (MDV) antibody titre, c) *Pasteurella multocida* (PM) antibody titre, d) *Eimeria* parasitism resistance, e) cestodes parasitism resistance, f) body weight, g) body condition score (BCS). The solid yellow line represents the significance threshold. The line with squares represents the ratio of the genes within each pathway to the total number of genes in the pathway.

1. Infectious Bursal Disease Virus (IBDV) antibody titre


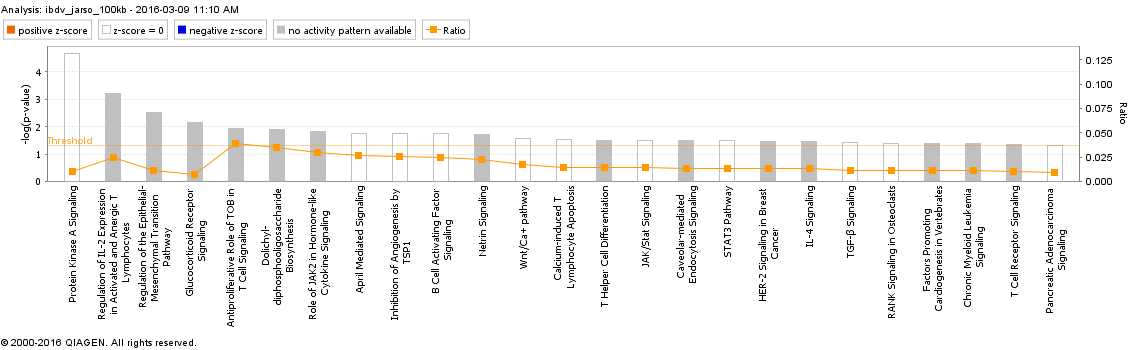


1. Marek’s Disease Virus (MDV) antibody titre


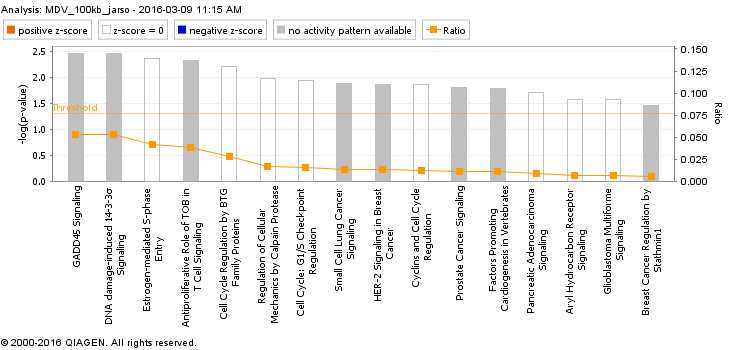


1. *Pasteurella multocida* (PM) antibody titre


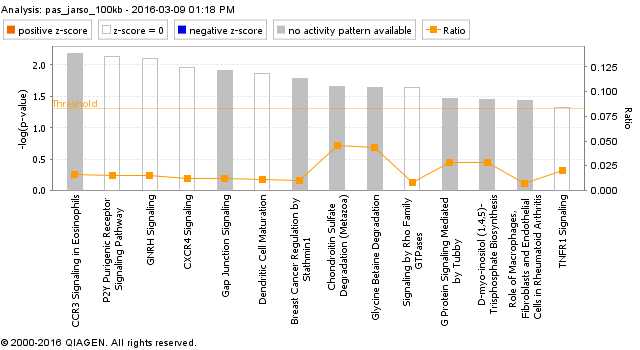


1. *Eimeria* parasitism resistance


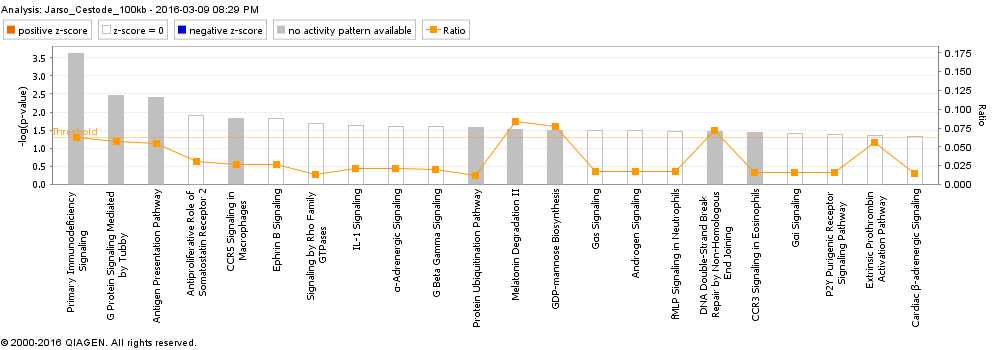


1. Cestodes parasitism resistance


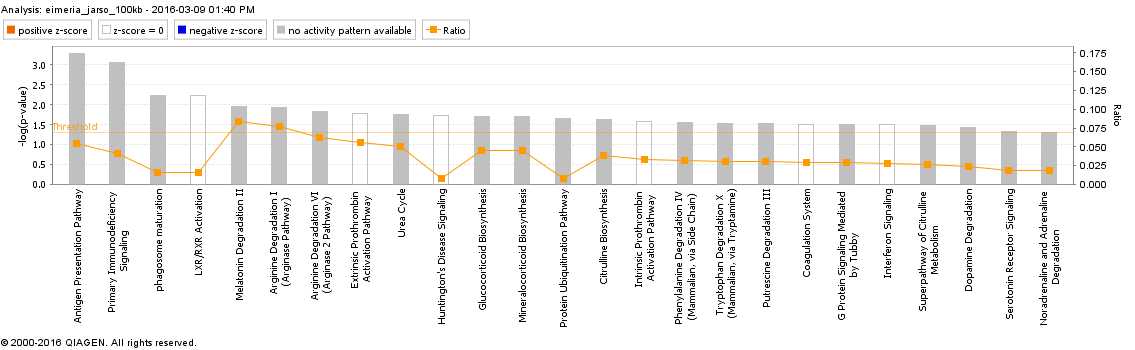


1. Body weight


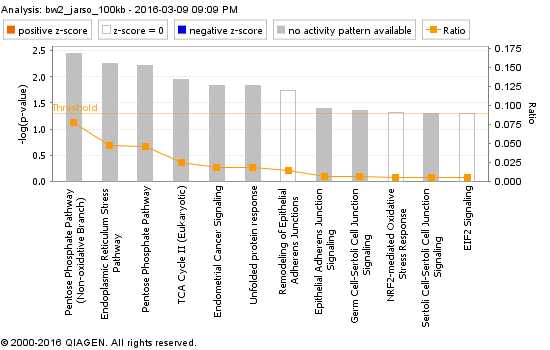


1. Body condition score (BCS)


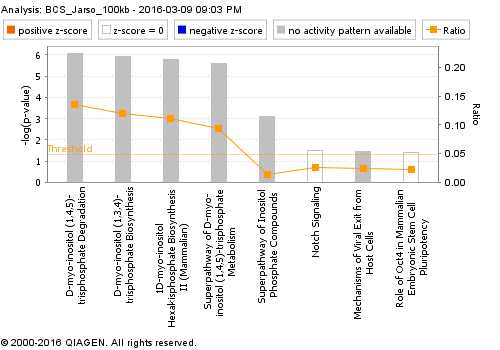


**Figure S5.** **Pathway analysis results using the IPA software for the Horro chickens**. The most highly represented canonical pathways of genes located at the candidate genomic regions for a) Infectious bursal disease virus (IBDV) antibody titre, b) Mareks’ disease virus (MDV) antibody titre, c) *Salmonella enterica* serovar Galinarum (SG) antibody titre, d) *Pasteurella multocida* (PM) antibody titre e) cestodes parasitism resistance, f) body weight. The solid yellow line represents the significance threshold. The line with squares represents the ratio of the genes within each pathway to the total number of genes in the pathway.

1. Infectious Bursal Disease Virus (IBDV) antibody titre


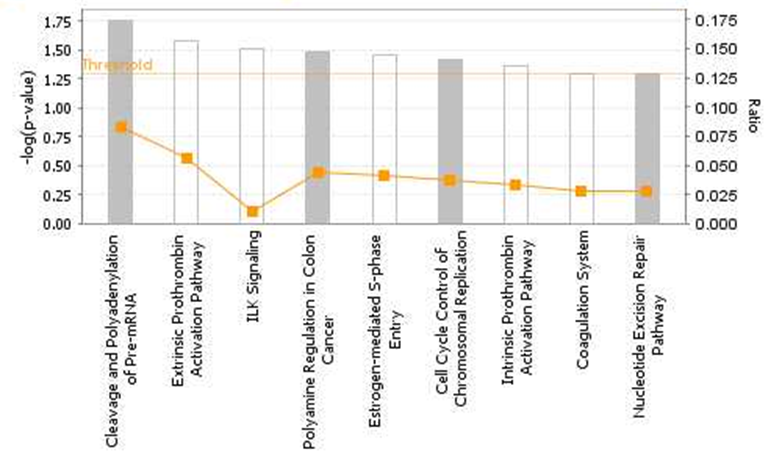


1. Mareks’ Disease Virus (MDV) antibody titre


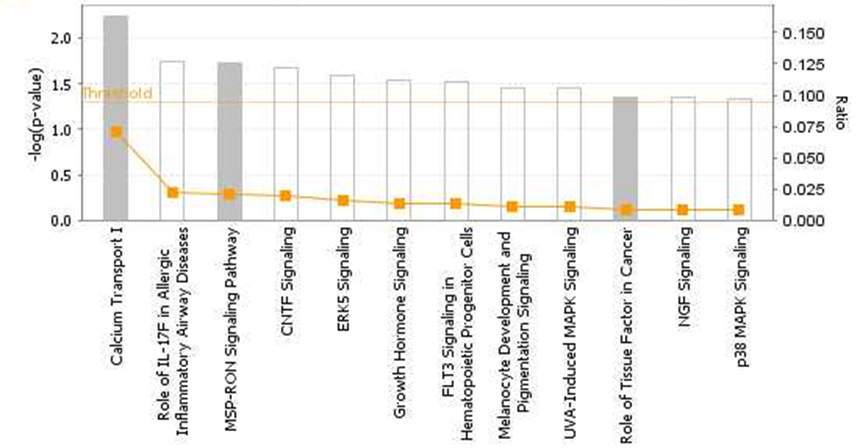


1. *Salmonella enterica* serovar Gallinarum (SG) antibody titre


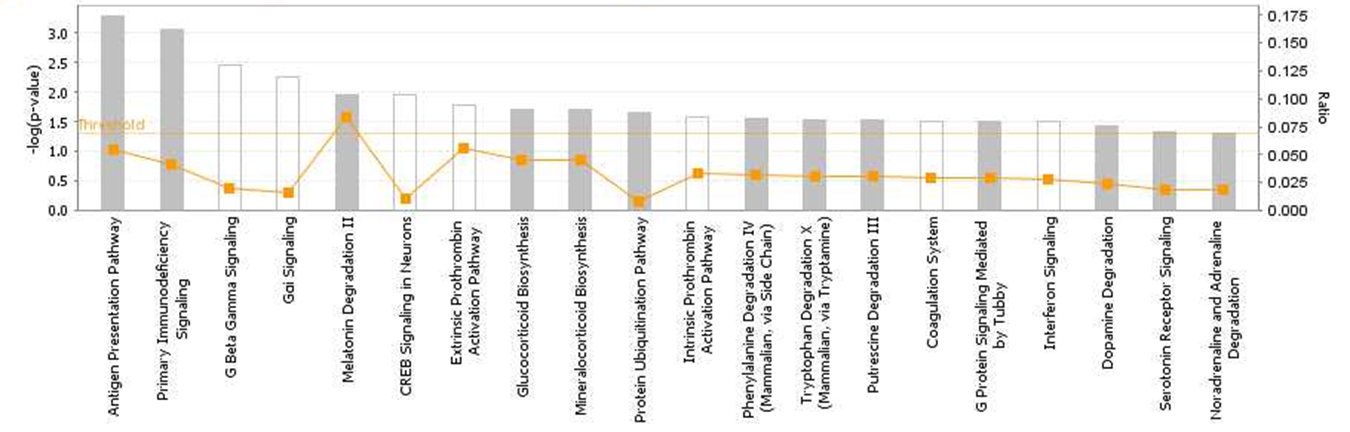


1. *Pasteurella multocida* (PM) antibody titre


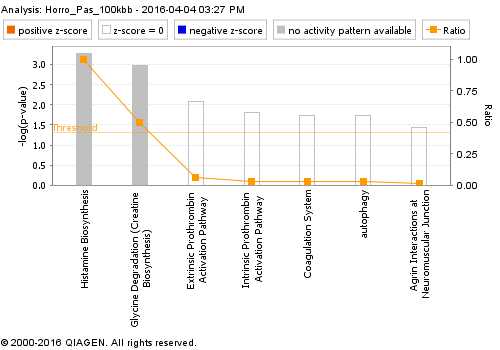


1. Cestodes parasitism resistance


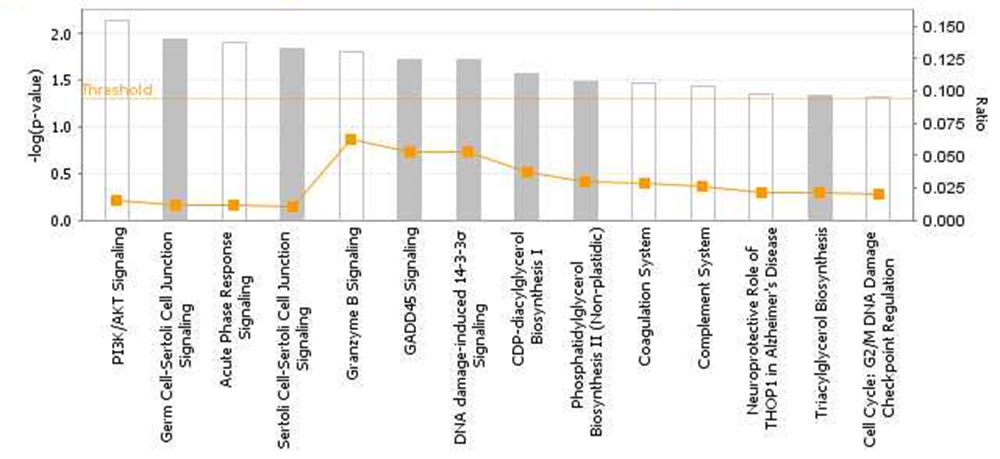


1. Body weight


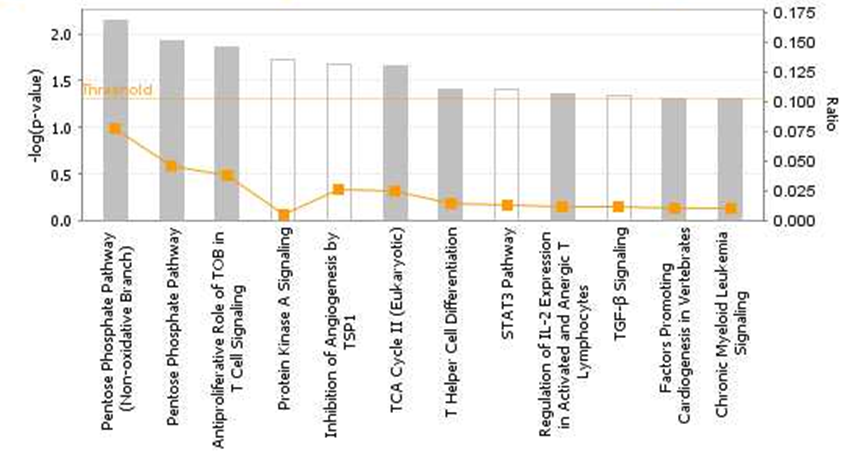

Supplement: Supplementary file 7 — 10.1186/s12711-016-0252-7 Pathway analysis results using the IPA software for the Jarso (Figure S4) and Horro (Figure S5) chickens. Description (Figure S4): The most highly represented canonical pathways for the genes located in the candidate genomic regions for (a) infectious bursal disease virus (IBDV) antibody titre, (b) Mareks’ disease virus (MDV) antibody titre, (c) Pasteurella multocida (PM) antibody titre, (d) Eimeria parasitism resistance, (e) cestodes parasitism resistance,(f) body weight, (g) body condition score (BCS). The solid yellow line represents the significance threshold. The line with squares represents the ratio of the genes within each pathway to the total number of genes in the pathway. Description (Figure S5): The most highly represented canonical pathways for the genes located in the candidate genomic regions for (a) infectious bursal disease virus (IBDV) antibody titre, (b) Mareks’ disease virus (MDV) antibody titre, (c) Salmonella enterica serovar Galinarum (SG) antibody titre, (d) Pasteurella multocida (PM) antibody titre (e) cestodes parasitism resistance, (f) body weight. The solid yellow line represents the significance threshold. The line with squares represents the ratio of the genes within each pathway to the total number of genes in the pathway. [file 12711_2016_252_MOESM7_ESM.docx]
