# Supplementary material for: Genome-wide association studies of immune, disease and production traits in indigenous chicken ecotypes
Source: Genet Sel Evol. 2016 Sep 29;48:74. doi: 10.1186/s12711-016-0252-7 (PMC5041578; doi:10.1186/s12711-016-0252-7)

**Additional File 8: Figure S6 and Figure S7.**

**Figure S6. Network analysis results using IPA software for the Jarso chickens.** The networks illustrate the molecular interactions between products of genes selected from candidate genomic regions for Infectious bursal disease virus (IBDV) antibody titre (Network A and B); Mareks’ disease virus (MDV) antibody titre (C); *Salmonella enterica* serovar Galinarum (SG) antibody titre (D); *Eimeria* parasitism resistance (E and F); cestodes parasitism resistance (G and H); body condition score (BCS, I). Solid and dashed arrows represent direct and indirect interactions, respectively. The white colour indicates gene products added to the IPA analysis because of their interaction with the target gene products.

1. Network related with organismal injury and abnormalities, and tissue morphology (IPA score 26) illustrate molecular interactions between products of candidate genes selected from the candidate regions for antibody titres to Infectious Bursal Disease Virus (IBDV)


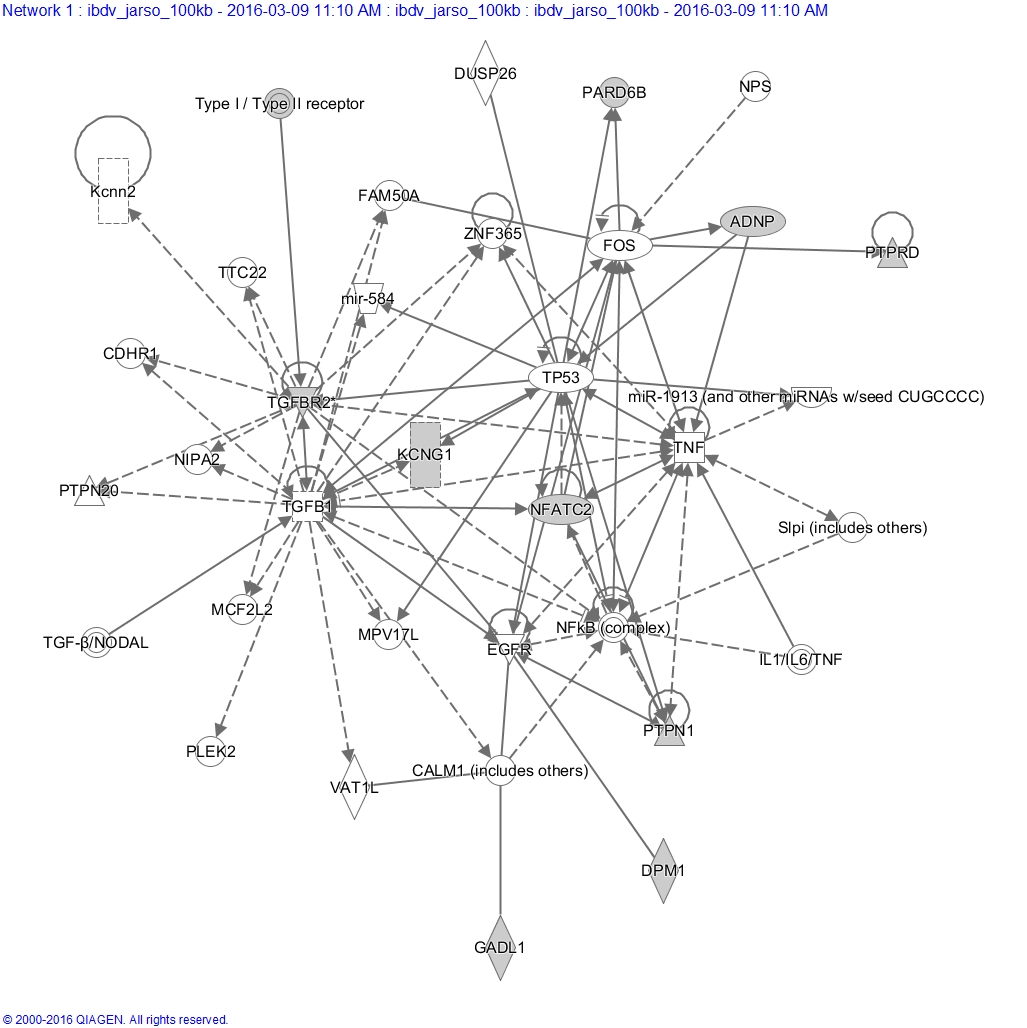


1. Network related with lymphoid tissue structure and development, and organ and tissue morphology cellular assembly and organisation (IPA score 33) illustrate molecular interactions between products of candidate genes selected from the candidate regions for antibody titres to Infectious Bursal Disease Virus (IBDV)


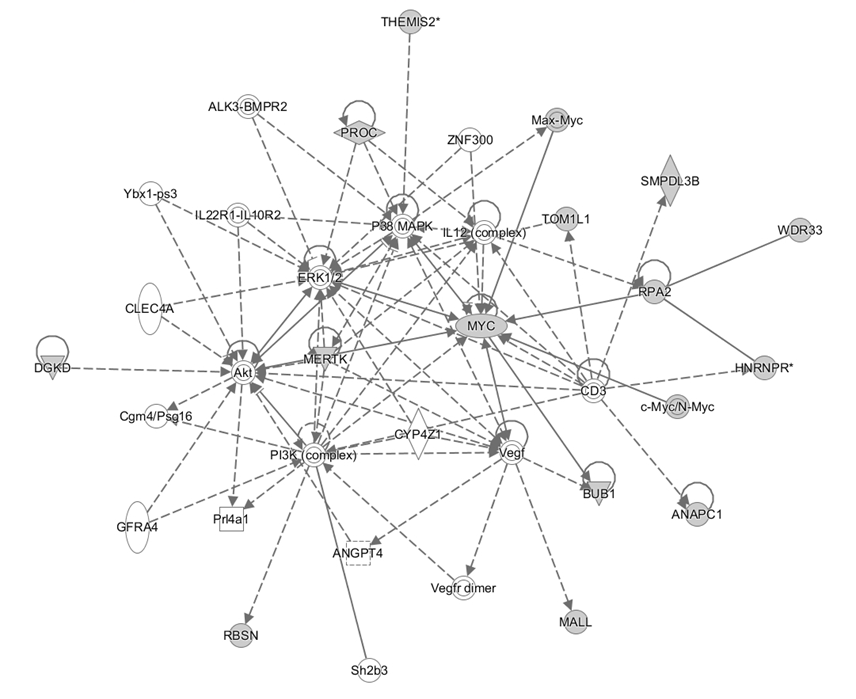


1. Network related with inflammatory disease, and connective tissue disorder (IPA score 15) illustrate molecular interactions between products of candidate genes selected from the candidate regions for antibody titres to Mareks’ DiseaseVirus (MDV)


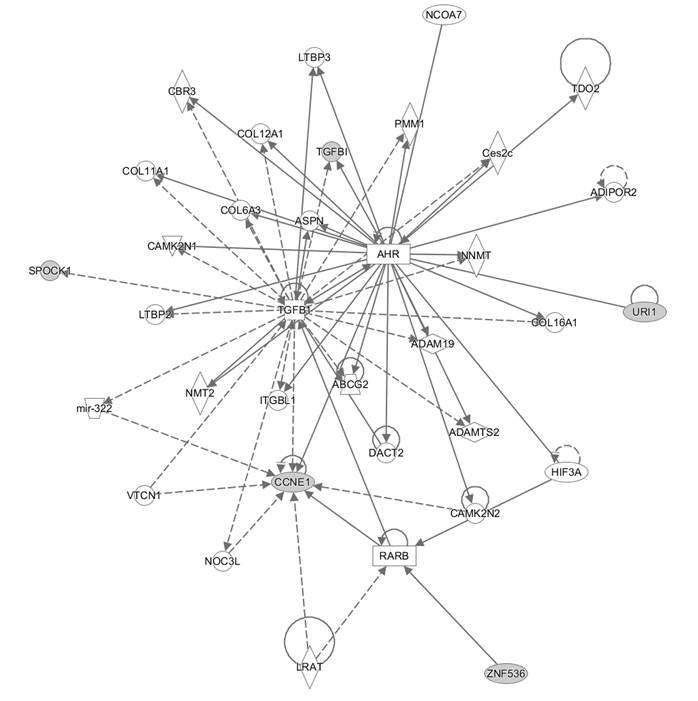


1. Network related with cellular assembly and organisation, immunological disease and hereditary disorder (IPA score 37) illustrate molecular interactions between products of candidate genes selected from the candidate regions for antibody titres to *Salmonella enterica* serovar Galinarum (SG)


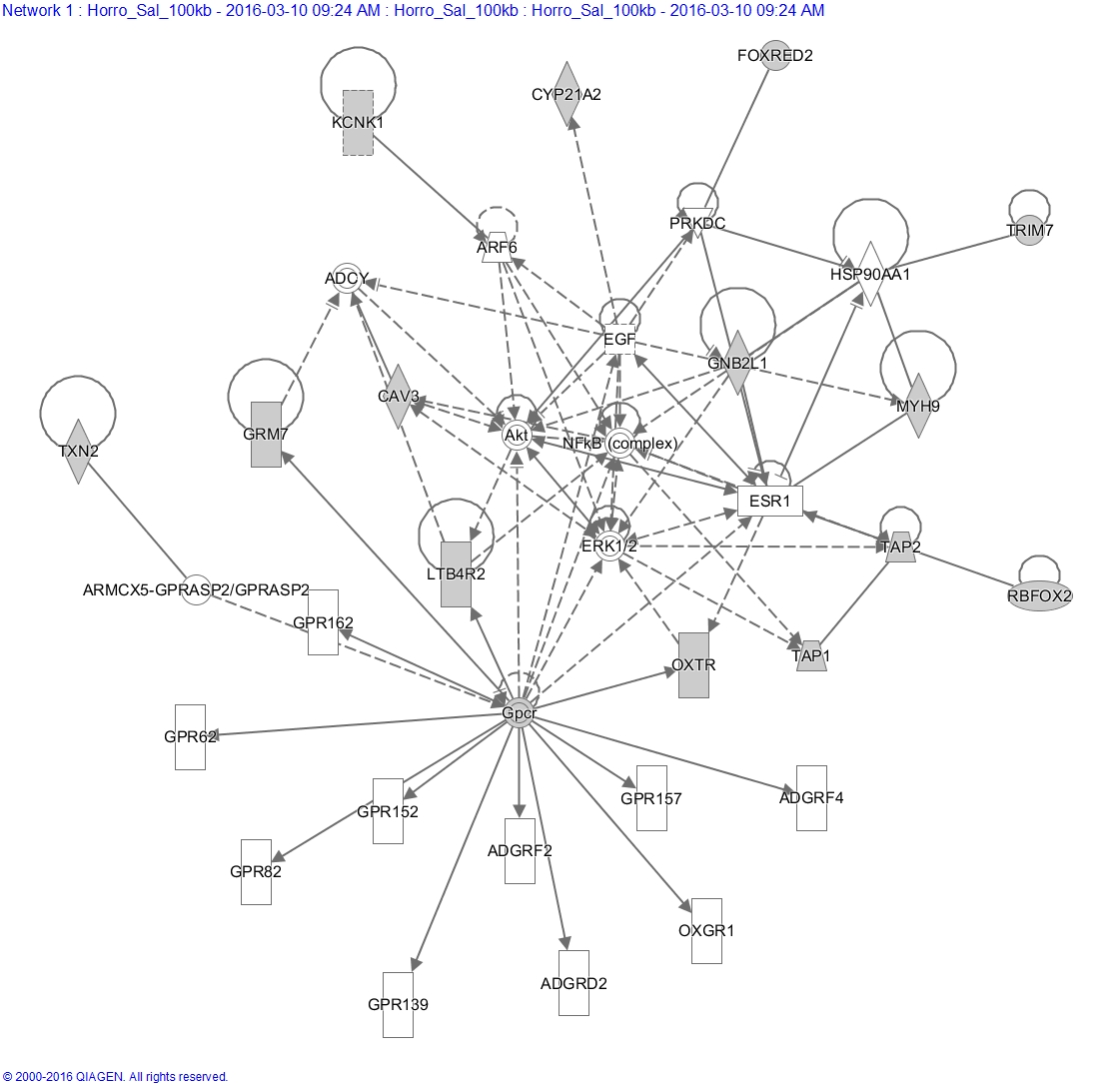


1. Network related with immunological disease (IPA score 24) illustrate molecular interactions between products of candidate genes selected from the candidate regions for *Eimeria* resistance


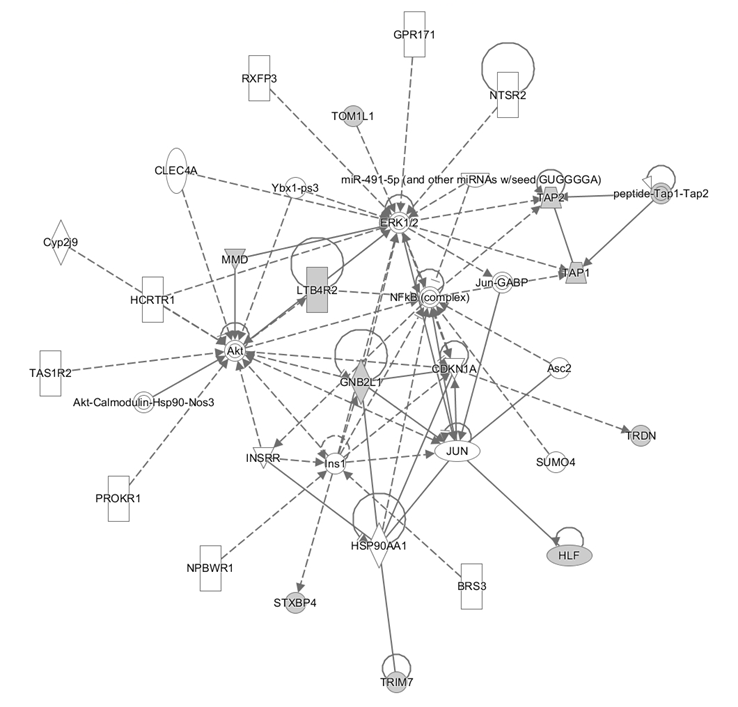


1. Network related with cell death and survival, connective tissue disorder, cell to cell signalling and interaction (IPA score 18) illustrate molecular interactions between products of candidate genes selected from the candidate regions for *Eimeria* resistance


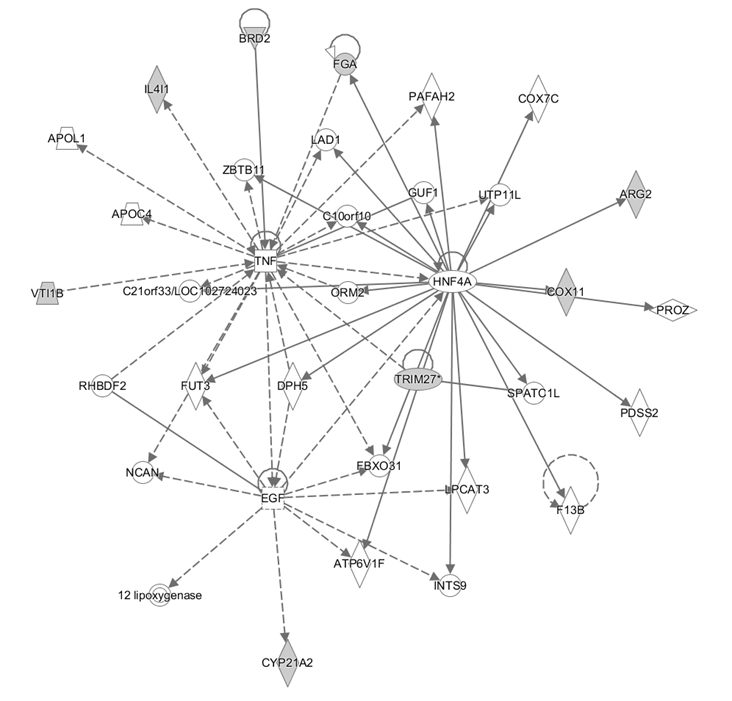


1. Network related with organismal injury and abnormalities, and cellular compromise (IPA score 19) illustrate molecular interactions between products of candidate genes selected from the candidate regions for cestodes resistance


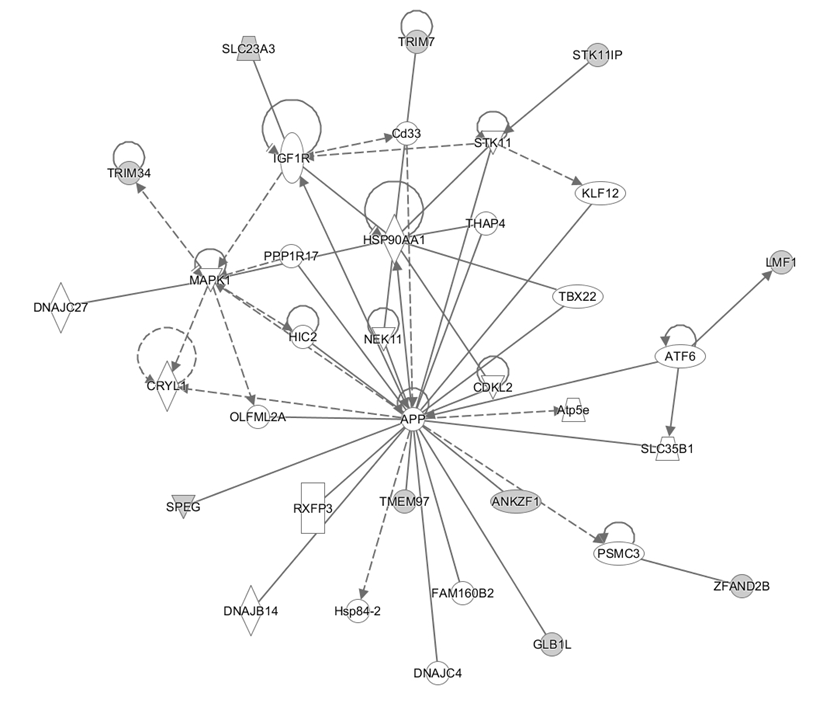


1. Network related with organismal injury and abnormalities, organismal functions and cell cycle (IPA score 17) illustrate molecular interactions between products of candidate genes selected from the candidate regions for cestodes resistance


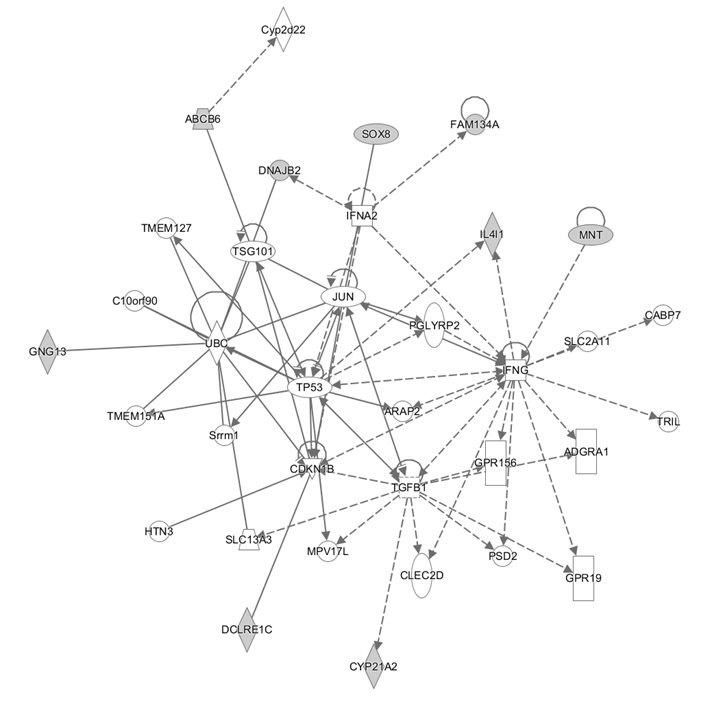


1. Network related with carbohydrate metabolism, lipid metabolism, small molecule biochemistry (IPA score 28) illustrate molecular interactions between products of candidate genes selected from the candidate regions for body condition score (BCS).


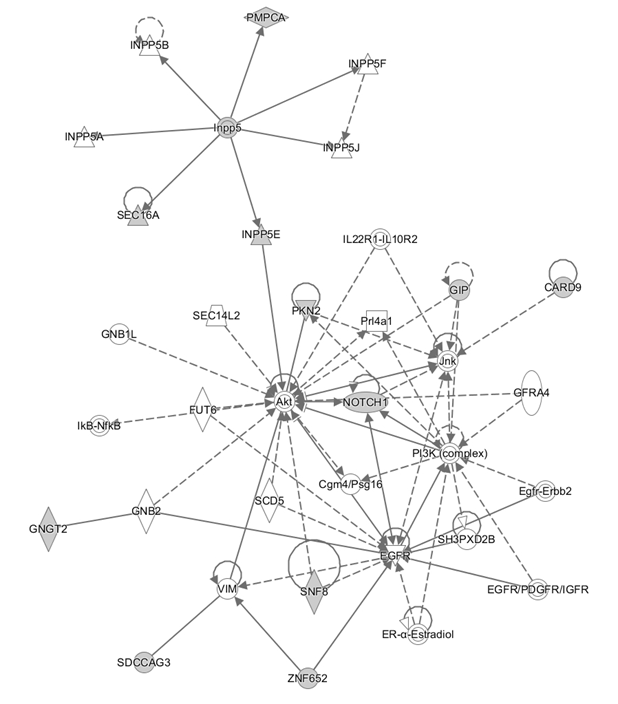


**Figure S7.** **Network analysis results using IPA software for the Horro chickens.** The networks illustrate molecular interactions between products of genes selected from the candidate genomic regions for *Salmonella enterica* serovar Galinarum (SG) antibody titre (Network A); for *Pasteurela multocida* (PM) antibody titre (B); for *cestodes* parasitism resistance (C, D and E); for body weight (F). Solid and dashed arrows represent direct and indirect interactions, respectively. The white colour indicates gene products added to the IPA analysis because of their interaction with the target gene products.

1. Network related with haematological disease, immunological disease and cancer (IPA score 13) illustrate molecular interactions between products of candidate genes selected from the candidate regions for antibody titres to *Salmonella enteritidis* serovar Gallinarum (SG)


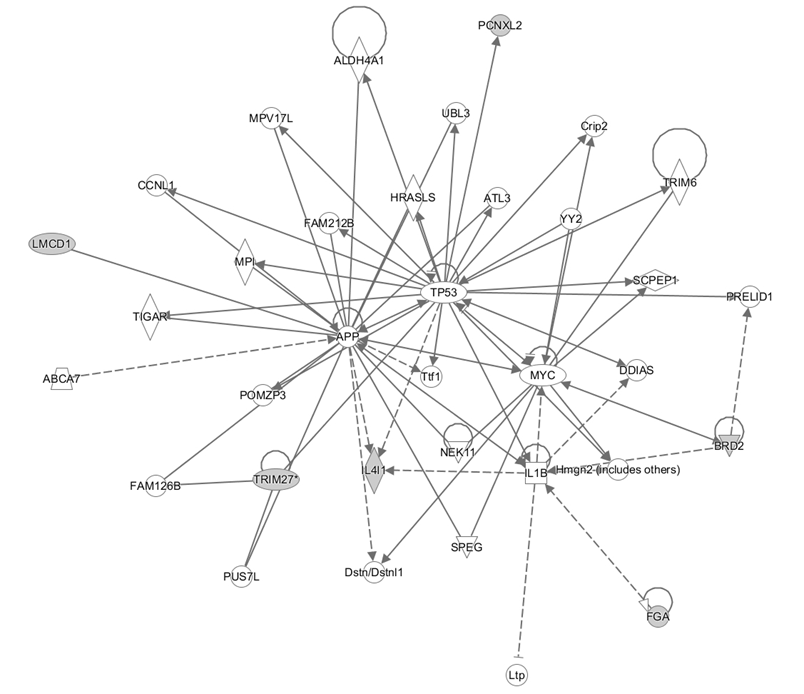


1. Network related with cell cycle, connective tissue development and function, free radical scavenging (IPA score 30) illustrate molecular interactions between products of candidate genes selected from the candidate regions for antibody titres to *Pasteurella multocida.*


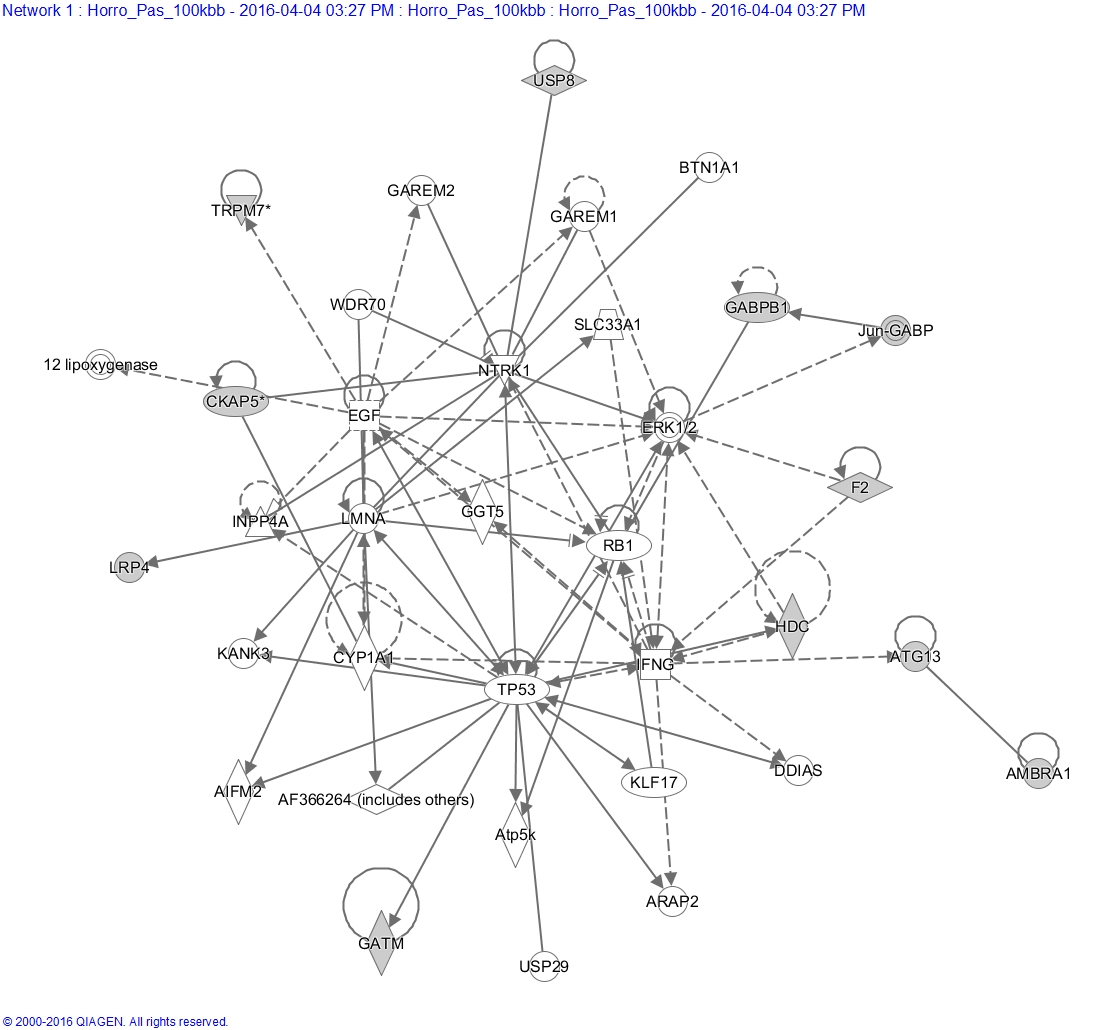


1. Network related with immunological disease and hereditary disorder (IPA score 67) illustrate molecular interactions between products of candidate genes selected from the candidate regions for *cestodes* resistance.


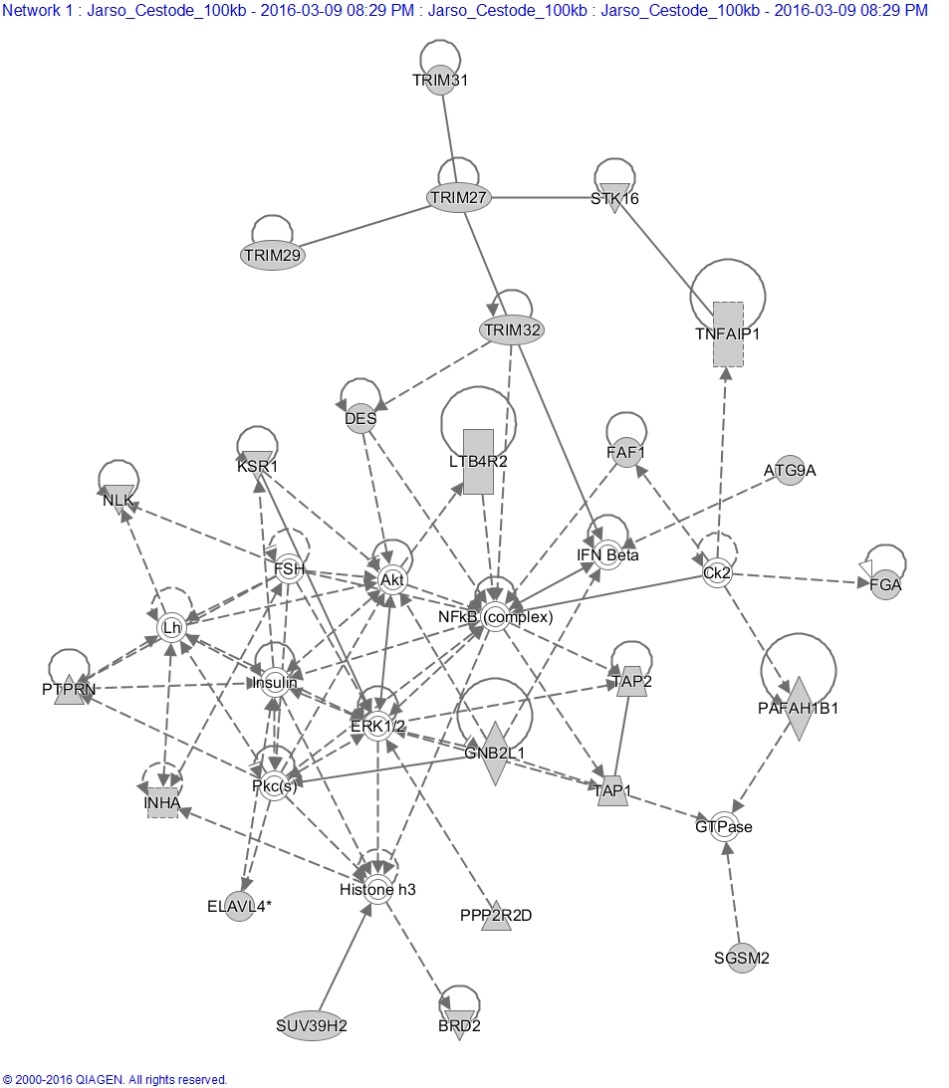


1. Network related with molecular transport, small molecule biochemistry, amino acid metabolism (IPA score 21) illustrate molecular interactions between products of candidate genes selected from the candidate regions for *cestodes* resistance


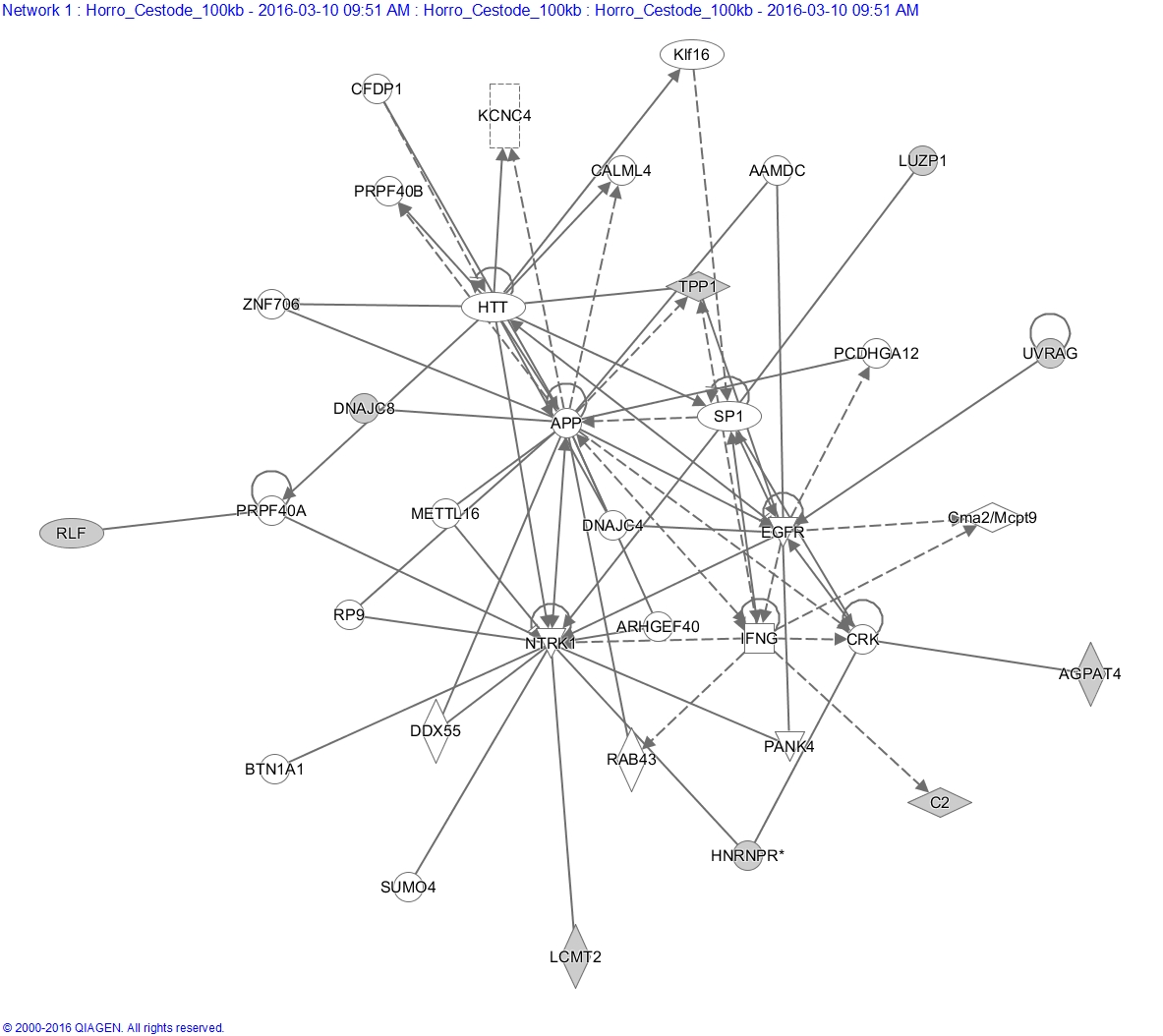


1. Network related with humoral immune response, protein synthesis and cellular function and maintenance (IPA score 18) illustrate molecular interactions between products of candidate genes selected from the candidate regions for *cestodes* resistance


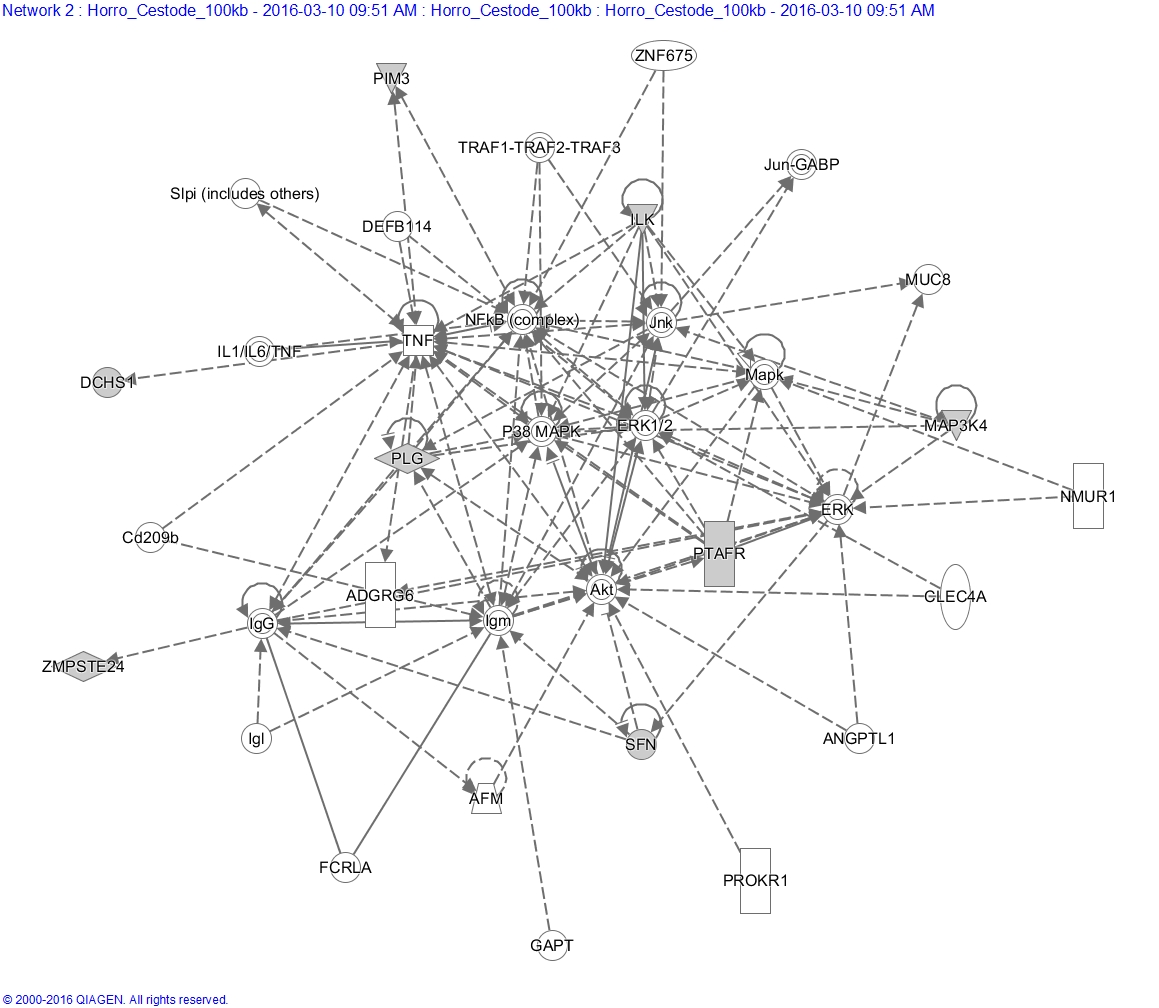
E) Network related with cell cycle and cellular development (IPA score 31) illustrate molecular interactions between products of candidate genes selected from the candidate regions for body weight.


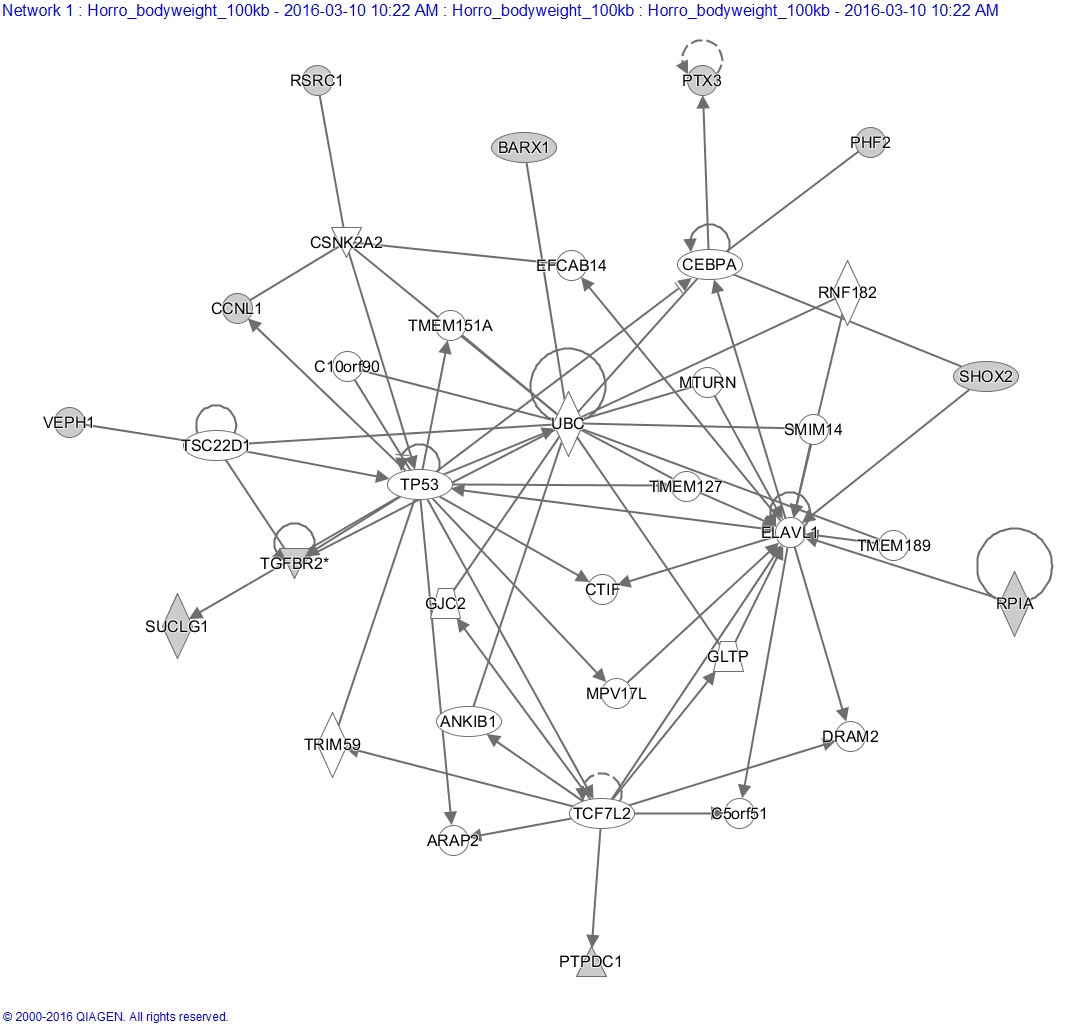

Supplement: Supplementary file 8 — 10.1186/s12711-016-0252-7 Network analysis results using IPA software for the Jarso (Figure S6) and Horro (Figure S7) chickens. Description (Figure S6): The networks illustrate the molecular interactions between products of genes selected from candidate genomic regions for infectious bursal disease virus (IBDV) antibody titre (networks A and B); Mareks’ disease virus (MDV) antibody titre (C); Eimeria parasitism resistance (E and F); cestodes parasitism resistance (G and H); body condition score (BCS, I). Solid and dashed arrows represent direct and indirect interactions, respectively. The white colour indicates gene products added to the IPA analysis because of their interaction with the target gene products. Description (Figure S7): The networks illustrate the molecular interactions between products of genes selected from the candidate genomic regions for Salmonella enterica serovar Galinarum (SG) antibody titre (network A); for Pasteurela multocida (PM) antibody titre (B); for cestodes parasitism resistance (C , D and E); for body weight (F). Solid and dashed arrows represent direct and indirect interactions, respectively. The white colour indicates gene products added to the IPA analysis because of their interaction with the target gene products. [file 12711_2016_252_MOESM8_ESM.docx]
